# Basis for lineage-determining pioneer factors targeting distinct repressed chromatin states

**DOI:** 10.1126/sciadv.adz7409

**Published:** 2026-01-09

**Authors:** Andrew Katznelson, Jingchao Zhang, Greg Donahue, Kenneth S. Zaret

**Affiliations:** ^1^ Institute for Regenerative Medicine, University of Pennsylvania, Philadelphia, PA 19104, USA.; ^2^ Epigenetics Institute, University of Pennsylvania, Philadelphia, PA 19104, USA.; ^3^ Cell and Molecular Biology Graduate Program, Perelman School of Medicine, University of Pennsylvania, Philadelphia, PA 19104, USA.

## Abstract

Pioneer transcription factors target transcriptionally silent chromatin, thereby enabling gene activation in development, regeneration, and cell reprogramming. However, silent chromatin is heterogeneous, varying in nucleosome stability, nucleosome compaction, and repressive histone modifications, and how pioneer factors may differentially overcome these different chromatin barriers is unknown. We systematically compared the chromatin targeting of 13 embryonic transcription factors and found that the DNA binding domain (DBD) type predicts whether a pioneer factor targets low-turnover nucleosomes in compact chromatin, dynamic nucleosomes in compact chromatin or functions as a nonpioneer factor targeting accessible chromatin. By contrast, non-DBD domains enable targeting of repressed chromatin marked by H3K9me3 or H3K27me3***.*** Fusions of different non-DBD segments of heterochromatin-targeting pioneer factors to the transcription factor SOX2 can expand binding of SOX2 target motifs within heterochromatin and improve cellular reprogramming. Our study unveils how different forms of silent chromatin are coordinately targeted by lineage-specifying factors.

## Teaser

Developmental transcription factors display a range of targeting H3K9me3 heterochromatin to open chromatin, for cell fate changes.

## INTRODUCTION

Pioneer transcription factors initiate changes in cell identity by their ability to target sites in transcriptionally silent, nuclease-resistant chromatin, enabling additional transcription factors and chromatin remodelers to bind and open the chromatin (*1*–*3*). Pioneer factors can bind targets embedded in low-turnover nucleosomes, reflecting their capacity to overcome stable chromatin barriers (*4*). The closed chromatin targeting ability of pioneer factors is conferred by recognition of DNA motifs, or partial motifs, on the nucleosome surface (*5*–*8*). Recent structural studies show that nucleosome binding by pioneer factors is facilitated by interactions with core histones, establishing that nucleosome displacement is not a prerequisite for targeting (*9*–*12*). Mutations in pioneer factors that perturb nucleosome binding and local chromatin opening, without impairing free DNA binding, can perturb developmental function (*13*, *14*). Since the initial finding that pioneer factors endow competence for the endoderm to activate a liver fate (*15*–*17*), pioneer factors have been found to initiate cell fate transitions in embryonic development, tissue regeneration, hormone-responsive cancers, and directed cell reprogramming (*18*, *19*). While a defining feature of pioneer factors is their ability to bind nucleosomal DNA, it remains unclear how different closed chromatin states influence pioneer factor binding (*2*). Transcriptionally silent chromatin states include so-called naïve or low-signal transcriptionally silent chromatin, enriched for linker histone (*20*), H3K27me3-marked domains repressed by the Polycomb machinery (*21*) and H3K9me3-marked domains repressed by HP1 (*22*). In this study, we address the initial step of pioneering, how transcription factors engage different closed chromatin states, and discover features by which developmental transcription factors target such different closed types to elicit cell fate changes.

## RESULTS

### DNA binding domain structure predicts pioneer factor nucleosome targeting

**Fig. 1.**
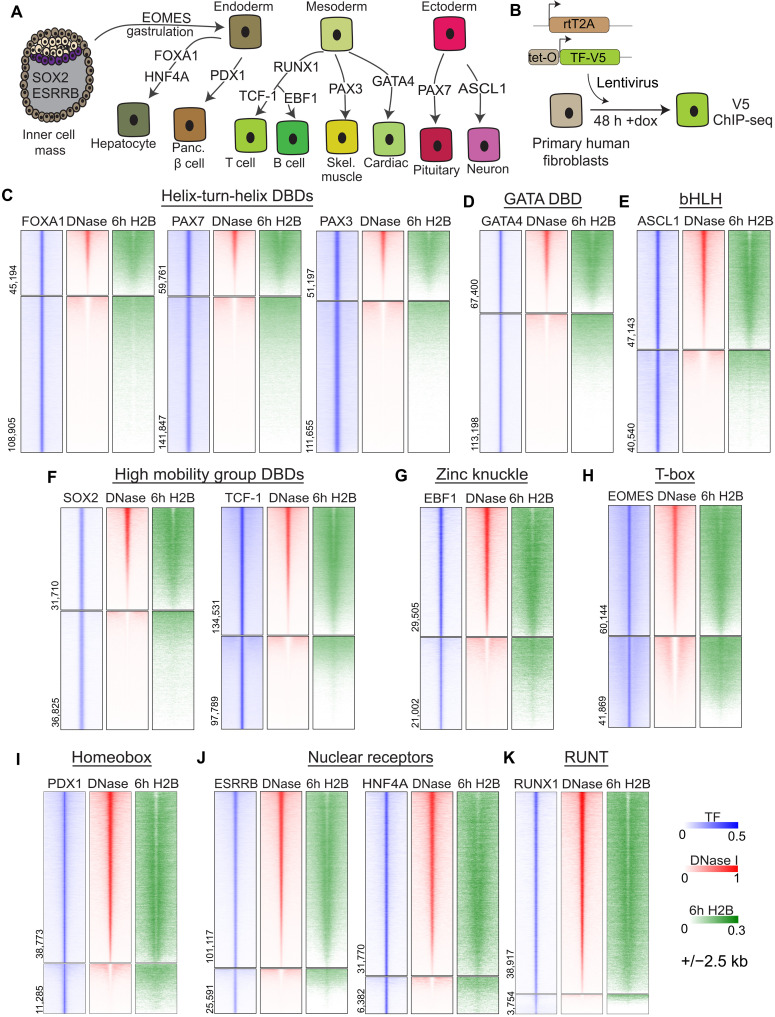
Transcription factors target high- and low-turnover, compacted chromatin. (**A**) Cartoon annotating a nonexhaustive list of developmental function of transcription factors in this study. h, hours. (**B**) Experimental design for lentiviral ectopic expression and ChIP-seq of tagged transcription factors. dox, doxycycline. (**C** to **K**) Preexisting chromatin accessibility and H2B turnover at TF-V5 ChIP-seq peaks after 48-hour ectopic expression of (C) helix-turn-helix transcription factors, (D) GATA transcription factor, (E) basic helix-loop-helix transcription factor, (F) high mobility group transcription factors, (G) zinc knuckle transcription factor, (H) T-box transcription factor, (I) homeobox transcription factor, (J) Nuclear receptor transcription factors, and (K) Runt transcription factor. bHLH, basic helix-loop-helix.

We systematically compared chromatin targeting by 13 developmental transcription factors that direct various stages of early mammalian development for their differential abilities to target silent chromatin after exogenous expression in primary human BJ fibroblasts (Fig. 1A). As these transcription factors have minimal or no detectable expression in BJ fibroblasts, we expressed each with a V5 tag at comparable protein levels for 48 hours using a Tet-On lentiviral vector (Fig. 1B and fig. S1, A to D). While transcription factor expression can vary between cell types, we previously found conditions where the lentiviral vector induces ~2- to 3-fold higher expression than endogenous FOXA1 and HNF4A in liver cells (*4*). Using SOX2, we find that it is expressed at approximately 2.5-fold of the normal protein in embryonic stem cells, placing our ectopic expression above endogenous levels but within a physiological range (fig. S1, E and F). We then performed chromatin immunoprecipitation sequencing (ChIP-seq) for the V5 antigen and identified sites where each factor targets the genome, including for three factors that we analyzed identically in a prior publication (table S1) (*4*, *23*). As expected, each factor targets its cognate motifs (fig. S2, A and B). We assessed the target sites by their chromatin accessibility in the fibroblasts before ectopic factor expression and ordered the transcription factors by their extent of targeting deoxyribonuclease (DNase)–accessible (open) or DNase-resistant (closed) chromatin (Fig. 1, C to J; upper and lower sections of heatmaps, respectively, fig. S3, A to C). While genomic motif positions are equivalently distributed between open and closed chromatin (fig. S3D), we observed a range of targeting to closed chromatin, from 70.6 to 8.7% (fig. S3E), consistent with there being a range of pioneering activity among developmental transcription factors.

Systematic Evolution of Ligands by Exponential Enrichment (SELEX) assays and cryo–electron microscopy studies showed that DNA binding domains (DBDs) from different transcription factor families bind in distinct ways to nucleosomes in vitro (*6*, *12*). The strongest closed chromatin-targeting pioneer factors, FOXA1, PAX7, and PAX3 (Fig. 1C and fig. S3E, yellow box), share DBDs with a helix-turn-helix structure and additional DNA-contacting segments (*24*, *25*). High mobility group factors SOX2 and TCF-1, helix-loop-helix-containing factors ASCL1 and EBF1, and GATA4 and EOMES display intermediate levels of closed chromatin targeting (Fig. 1, D to H) and would be characterized as pioneer factors. By contrast, despite their developmental importance, RUNX1, PDX1, and nuclear receptor proteins ESRRB and HNF4a all displayed weak closed chromatin targeting (Fig. 1, I to K). On the basis of total and proportional binding in closed chromatin, we conclude that at comparable expression levels, DBD types and nucleosome interaction abilities (*6*, *7*) correlate with whether a transcription factor binds chromatin as a pioneer or nonpioneer factor in vivo (fig. S3E).

We previously measured nucleosome turnover in chromatin by pulsing primary human fibroblasts with tagged histone H2B (H2B) for 6 hours (*4*). We chose to measure H2B turnover rather than H3 turnover as H2A-H2B dimers are assembled onto the H3-H4 core (*26*), thus mapping sites of new H2B integration most readily reflects dynamic turnover of nucleosomes (henceforth, 6h H2B). As expected, the DNase-accessible sites targeted by all the transcription factors are in regions of high H2B turnover (Fig. 1, C to K, top part of H2B heatmaps). We find low 6h H2B signals, i.e., low turnover, at most closed chromatin regions targeted by FOXA1, PAX7, PAX3, GATA4, SOX2, TCF1, and ASCL1 (Fig. 1, C to F, and fig. S4A). Most of the closed chromatin sites targeted by EBF1 and EOMES exhibited higher H2B turnover, particularly at the peak centers (Fig. 1, G and H, and fig. S4B), as did the low amounts of closed chromatin sites targeted by PDX1, ESRRB, HNF4A, and RUNX1 (Fig. 1, I to K, and fig. S4B). As an independent assessment, we found that the closed chromatin sites were resistant to high concentrations of micrococcal nuclease (MNase; fig. S4, C and D), confirming that the closed sites targeted in both high and low H2B turnover regions are enriched for nucleosomes. We conclude that most of the transcription factors classified as pioneers target low-turnover nucleosomes, although several prefer higher-turnover nucleosomes in DNase-resistant chromatin. Previously described nonpioneers, such as HNF4a and RUNX1 (*27*, *28*), primarily target DNase-accessible sites with high turnover nucleosomes.

We next explored sequence determinants that influence targeting of DNase-accessible or DNase-resistant chromatin. We used monaLisa (*29*), a computational tool that groups ChIP-seq binding sites by underlying DNase accessibility and searches for motifs differentially enriched in DNase-accessible or DNase-resistant sites (fig. S5A). While each transcription factor specifically targets its genomic motif, and not the motif of other assayed transcription factors (fig. S2B), we find that AP-1 sequences are highly and universally enriched in the most accessible, but not in the DNase-resistant, binding sites (fig. S5B). AP-1 elements are commonly found in motif analyses and stabilize transcription factor binding at a subset of sites (*30*), and our results show that this behavior is associated with active regulatory elements, not pioneered binding sites. By contrast, we find that the DNase-resistant bins are either enriched for the cognate transcription factor motif. We do not identify alternate motifs differentially associated with DNase-resistant sites (fig. S5B). This observation is consistent with previous analyses finding that higher motif density (*31*) or partial degenerate motifs (*5*) are required for pioneer factors to bind on the nucleosome. The absence of additional motifs associated specifically with closed chromatin suggests that pioneer factor binding is driven by cognate motif recognition, and not through interactions with a sequence-specific factor prepositioned on the chromatin.

### Pioneer factors use distinct modes of heterochromatin targeting

**Fig. 2.**
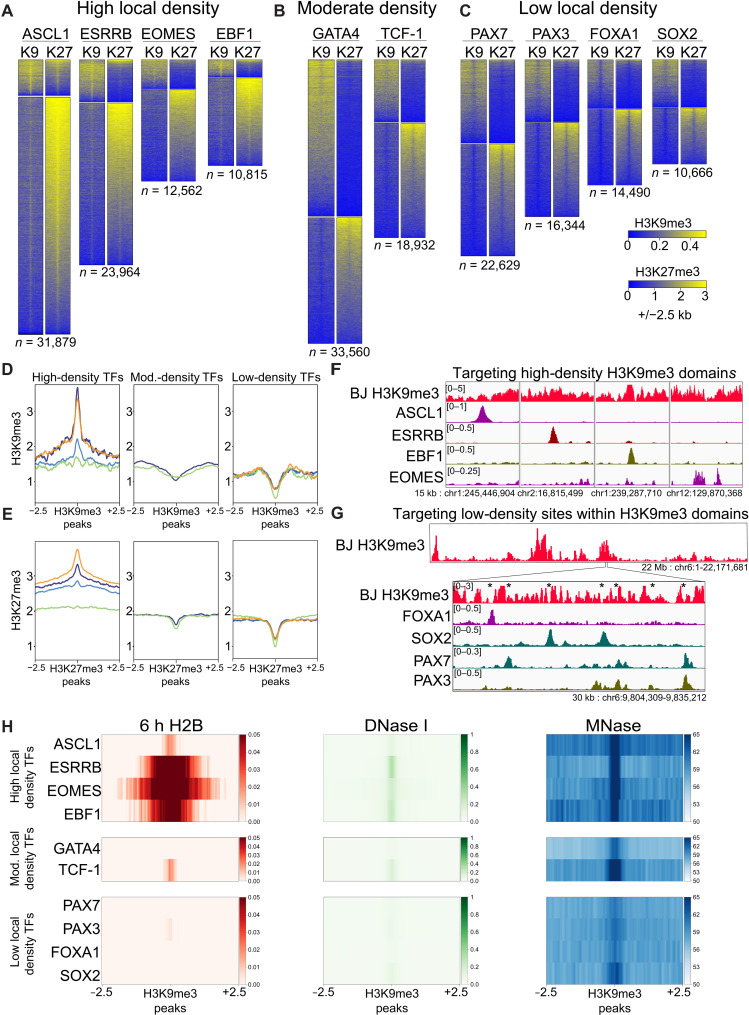
Differential heterochromatin targeting by pioneer factors. (**A** to **C**) Heatmaps showing preexisting H3K9me3 and H3K27me3 signals at pioneer factor binding sites located within H3K9me3 domains (top cluster) and H3K27me3 domains (bottom cluster), with domains annotated from (*33*). Factors are grouped by targeting to (A) high local heterochromatin density, (B) moderate local heterochromatin density, and (C) low local heterochromatin density. (**D** and **E**) Metaplots of (D) H3K9me3 signal under H3K9me3 pioneer factor binding sites (Mod., moderate) and (E) H3K27me3 signal under H3K27me3 pioneer factor binding sites, split by TF groupings defined in (A) to (C). Line colors indicate individual transcription factors. High-density TFs: ASCL1 (orange), EBF1 (purple), ESRRB (blue), and EOMES (green). Moderate-density TFs: GATA4 (purple) and TCF-1 (green). Low-density TFs: PAX7 (purple), PAX3 (blue), SOX2 (orange), and FOXA1 (green). Genome browser of pioneer factors targeting (**F**) high-density heterochromatin, (**G**) gaps in low-density heterochromatin domains. Stars indicate local heterochromatin gaps. (**H**) Heatmaps of 6h H2B, DNase I, and MNase-seq signal underlying H3K9me3 binding events.

While H3K9me3- and H3K27me3-marked heterochromatin have been shown to be refractory to targeting by certain pioneer factors (*23*, *32*–*35*), recent studies have found examples of transcription factors targeting such domains (*36*–*39*). Here, we intersected ectopic transcription factor binding sites with preexisting H3K9me3 and H3K27me3 domains and found that pioneer factors that strongly target closed chromatin include thousands of sites within broad heterochromatin domains, while factors that poorly target closed chromatin, PDX1, HNF4A1, and RUNX1, likewise poorly target heterochromatin (Fig. 2, A to C, and fig. S6, A and B). By assessing the levels of H3K9me3 and H3K27me3 around the heterochromatin binding events, we find that transcription factors target three distinct heterochromatin subtypes (Fig. 2, A to C, and fig. S6, C and D). ASCL1, ESRRB, EOMES, and EBF1 target heterochromatic regions where H3K9me3 and H3K27me3 are distinctly enriched beneath the transcription factor peaks compared to flanking regions, suggesting that the repressive chromatin state provides a favorable binding environment (Fig. 2, A, D, and F, and fig. S6, C and D). GATA4 and TCF-1 target heterochromatin sites that are as comparably enriched as flanking domains, indicating that these pioneers are not inherently blocked by heterochromatin (Fig. 2, B, D, and E, and fig. S6, C and D). While PAX7, PAX3, FOXA1, and SOX2 target within H3K9me3- and H3K27me3-marked heterochromatin domains, they target sites of local depletion, exploiting small gaps in broad domains (Fig. 2, C to E and G, and fig. S6, C and D). Thus, while all the pioneer factors tested target nucleosomal sites embedded in broad heterochromatin domains, local differences of H3K9me3 and H3K27me3 partition binding ability.

Our observation that ASCL1, ESRRB, EOMES, and EBF1 target highly enriched heterochromatin (Fig. 2, A, D, and E) as well as higher-turnover DNase-resistant chromatin (fig. S4, A and B) led us to ask whether this preference reflects targeting to more dynamic regions within heterochromatin domains. In highly enriched heterochromatin domains, we find that ASCL1 targets low-turnover nucleosomes, while ESRRB, EOMES, and EBF1 target low-accessible but highly dynamic, well-positioned nucleosomes (Fig. 2H, top). By contrast, PAX7, PAX3, FOXA1, and SOX2 target narrow windows within heterochromatin domains composed of naïve, DNase-resistant, low-turnover nucleosomes (Fig. 2H, bottom). The few H3K9me3 sites targeted by nonpioneers PDX1, HNF4A, and RUNX1 display a euchromatic H3K9me3 signature, with high histone turnover and chromatin accessibility (fig. S6E).

To understand a basis for chromatin differences that may be permissive or inhibitory to heterochromatin targeting, we assessed enrichment of H2A.Z and H3.3. These histone variants, in contrast to canonical core histones, are expressed throughout interphase and thus may reflect a more dynamic heterochromatic state (*40*, *41*). The factors that target heterochromatin-rich regions with higher-turnover nucleosomes preferentially target H2A.Z and H3.3, with ESRRB showing the strongest preference, consistent with its targeting of a more dynamic heterochromatin subset and DNase-accessible fraction (Figs. 1J and 2H and fig. S7, A and B). We conclude that transcription factors show distinct abilities to target an array of chromatin states, from highly enriched heterochromatin containing either stable or dynamic nucleosomes, stable nucleosomes lacking heterochromatin marks, or unstable nucleosomes lacking heterochromatin marks, suggesting that pioneer factors with different silent chromatin targeting preferences cooperate to elicit changes in gene expression programs.

### Non-DBD domains transfer heterochromatin targeting ability

**Fig. 3.**
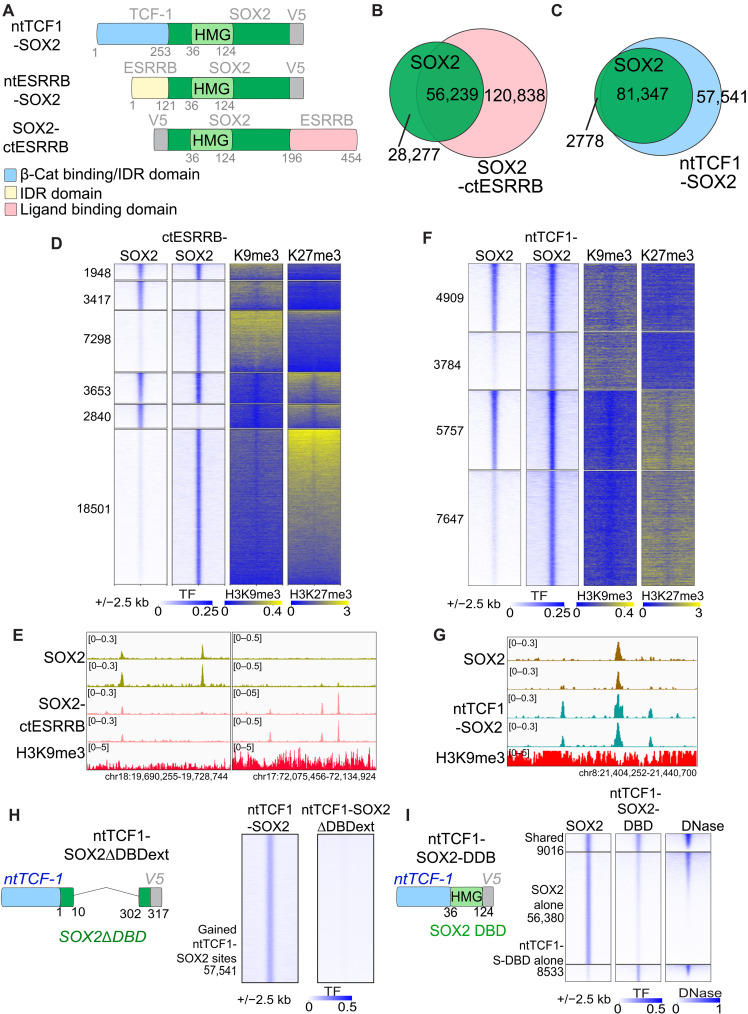
Hybrid SOX2 pioneer factors improve heterochromatin targeting via non-DBD protein domains. (**A**) Design of hybrid SOX2 fusion proteins. Numbers indicate amino acid positions corresponding to the full-length parental factors for the N- or C-terminal domains fused to SOX2, as well as the boundaries of the SOX2 HMG domain. (**B**) Venn diagram of SOX2 and SOX2-ctESRRB targeting events. (**C**) Venn diagram of SOX2 and ntTCF1-SOX2 targeting events. (**D**) Heatmap of preexisting H3K9me3 and H3K27me3 at SOX2-ctESRRB heterochromatin target events. (**E**) Genome browser of rerouted binding of SOX2-ctESRRB, with lost (left side) and gained sites (right side), biological replicate tracks shown. (**F**) Heatmap of preexisting H3K9me3 and H3K27me3 at ntTCF1-SOX2 heterochromatin target events. (**G**) Genome browser of expanded binding of ntTCF1-SOX2 in heterochromatin, biological replicate tracks shown. (**H**) Design (left) and heatmap (right) of assessment of ntTCF1-SOX2∆DBDext in chromatin binding. (**I**) Design (left) and heatmap (right) of assessment of ntTCF1-SOX2-DBD in DNase-resistant chromatin targeting.

Non-DBD domains influence transcription factor nuclear mobility and target choice, including within compact chromatin (*42*–*44*), and DBD-alone truncations of pioneer factors are deficient in binding closed chromatin sites (*4*, *45*). To test how non-DBD domains affect pioneer factor targeting of heterochromatin, we fused SOX2 to non-DBD domains from transcription factors that robustly target H3K9me3- and H3K27me3-marked heterochromatin. First, we fused SOX2 to the N terminus of TCF-1, without its HMG box DBD (ntTCF1-SOX2) (Fig. 3A and fig. S8A), since the N terminus harbors segments that facilitate chromatin binding (*46*). We also fused SOX2 to either the N- or C-terminal non-DBD domains of ESRRB (ntESRRB-SOX2, SOX2-ctESRRB) (Fig. 3A and fig. S8A), given ESRRB’s strong targeting of dynamic subsets of H3K9me3 and H3K27me3 (Fig. 2A) and that it functions alongside SOX2 during pluripotency specification (*36*, *37*). Western blot analyses after 48-hour transductions showed that the SOX2-ctESRRB protein level per cell was diminished compared to wild-type SOX2 (fig. S8B), apparently due to the ctESRRB domain’s intrinsic instability (fig. S8C) (*47*). Despite that, the SOX2-ctESRRB hybrid targets more sites than the ntTCF1-SOX2 hybrid (Fig. 3, D and F), and thus, we compared their activities.

While the ntESRRB-SOX2 fusion did not affect SOX2 targeting (fig. S8D), the ntTCF1-SOX2 and SOX2-ctESRRB fusions greatly expanded chromatin targeting. However, SOX2-ctESRRB notably diverts SOX2 away from many of its normal targets (66.3% retained peaks, 141% gained peaks; Fig. 3B), with most new targeting events in DNase-accessible chromatin (fig. S9A). By contrast, the ntTCF1-SOX2 hybrid expands SOX2 targeting while preserving wild-type SOX2 targeting events (97% retained peaks, 68.2% gained peaks; Fig. 3C), with most new targets in DNase-resistant chromatin (fig. S9B). Compared to wild-type SOX2, both hybrids targeted thousands more sites with H3K9me3 and H3K27me3 (Fig. 3, D to G), with SOX2-ctESRRB failing to target heterochromatic sites with low H3.3 and H2.AZ, reminiscent of wild-type ESRRB factors propensity to bind heterochromatin sites enriched for the histone variants (fig. S9, C to F). Since both hybrid SOX2 proteins target distinct sites with chromatin states similar to those targeted by wild-type ESRRB or TCF-1, we conclude that non-DBDs influence engagement with different forms of heterochromatin. Motif analysis of wild-type and hybrid peak sets show that both hybrids target SOX2 motifs, with SOX2-ctESRRB targeting a higher fraction of AP-1 active elements, consistent with its targeting a more accessible chromatin state (figs. S5B, S9B, and S10, A and B). Thus, while DBD domains dictate the sequence specificities of pioneer factor targets, non-DBDs can direct targeting to chromatin states, which is transferable.

To distinguish the relative contribution of the SOX2 DBD and non-DBD domains to the expanded chromatin targeting observed in SOX2 hybrids, we fused the N-terminal TCF1 domain either to a SOX2 mutant carrying an extended deletion encompassing the DBD and adjacent non-DBD regions (ntTCF1-SOX2∆DBDext) or to the SOX2 DBD alone (ntTCF1-SOX2-DBD) (Fig. 3, H and I, and fig. S11A). In the absence of SOX2’s DBD and adjacent non-DBD, the N-terminal TCF-1 domain did not specifically bind chromatin (Fig. 3H and fig. S11B), in contrast to a recent study finding that non-DBD domains of yeast transcription factors are sufficient for specific chromatin binding (*42*). We previously found that non-DBD domains of SOX2 are required for the DBD to efficiently bind DNase-resistant chromatin (*4*). When fused to just the SOX2 DBD, the N-terminal TCF1 domain expanded targeting in accessible chromatin but was not sufficient to rescue the loss of SOX2’s non-DBD domains for targeting closed chromatin (Fig. 3I). Thus, different non-DBDs stabilize binding to distinct chromatin states, and the full-length SOX2 is necessary for the enhanced heterochromatin targeting previously observed with the TCF-1 hybrid (Fig. 3, F and G). Truncations of the TCF-1 N terminus fused to SOX2 showed that various portions of the TCF1 N terminus partially contribute to expand SOX2 binding (fig. S11, C to E), akin to the partially redundant function of non-DBDs in chromatin binding of yeast transcription factors (*42*) and consistent with these domains’ functional role in T cell differentiation (*46*).

### Non-DBD domains modulate pioneer factor binding affinity across the genome

**Fig. 4.**
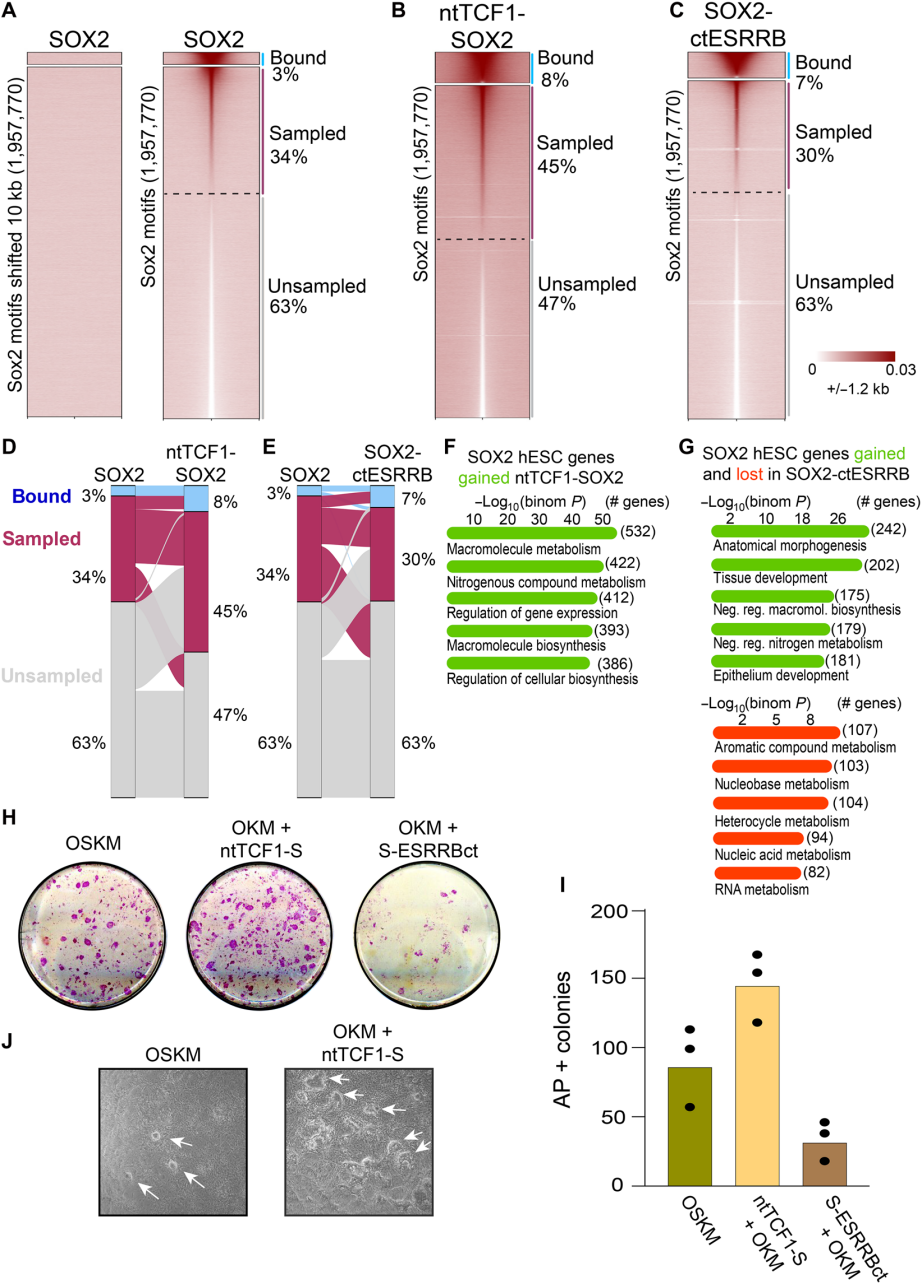
Non-DBD domains control hybrid SOX2 motif sampling, affinity, and cellular reprogramming ability. (**A**) Chromatin binding, sampling of SOX2 over Sox2 motifs (right) or SOX motifs shifted 10 kb as putative background regions (left). (**B** and **C**) Chromatin binding, sampling of (B) ntTCF1-SOX2 and (C) SOX2-ctESRRB over Sox2 motifs. (**D** and **E**) Alluvial diagram showing shifts of motif binding and sampling from (D) SOX2 to ntTCF1-SOX2 and (E) SOX2 to SOX2-ctESRRB. (**F**) Gene ontology analysis of target sites bound by SOX2 in human embryonic stem cells (hESCs) and ntTCF1-SOX2 in hBJ fibroblasts but not wild-type SOX2 in hBJ fibroblasts. (**G**) Gene ontology analysis of target sites bound by SOX2 in hESCs and SOX2-ctESRRB in hBJ fibroblasts but not wild-type SOX2 in hBJ fibroblasts (left) and target sites bound by SOX2 in hESCs and wild-type SOX2 but not SOX2-ctESRRB in hBJ fibroblasts. (**H**) Alkaline phosphatase (AP) staining of iPSC colonies at day 9 of reprogramming. (**I**) Quantification of AP staining. (**J**) Pluripotent colonies at day 14 after doxycycline removal.

Sites gained by the hybrid SOX2 proteins show a weak binding signal by wild-type SOX2 (Fig. 3, D and F; see low SOX2 signal in hybrid gained binding events), reminiscent of transient chromatin “sampling,” or low-frequency binding of motifs below the peak-calling threshold (*4*, *23*). We thus hypothesized that the hybrid non-DBD domains stabilize SOX2 on low-frequency targets. To map the potential SOX2 cistrome, we identified genome-wide instances of SOX2 motifs and found that while SOX2 only shows strong ChIP-seq enrichment 3% of its motifs, i.e., at called peaks, SOX2 displays specific, low-level sampling across 34% of its 1,957,770 motifs (Fig. 4A, right, and fig. S12, A and B). The specificity of the signals is demonstrated by no sampling seen at sites 10 kb upstream of the genomic SOX2 motifs (Fig. 4A, left, and fig. S12, B and C). The hybrid factors specifically bind and sample the same SOX2 motif set, with each factor increasing the total motifs bound (ntTCF1-SOX2: 8%, ctESRRB-SOX2: 7%; Fig. 4, B and C, and fig. S12, B and C). However, while the TCF1 N terminus increases the motifs sampled compared to wild-type SOX2, adding the ESRRB C terminus impedes sampling (45 and 30% of motifs sampled, respectively) (Fig. 4, B and C). Nearly all hybrid-gained binding originates from motifs sampled by SOX2 and vice versa for motifs that lose binding (Fig. 4, D and E, and fig. S12D). To identify specific chromatin states that associate with different binding or sampling behaviors, we trained a fibroblast-specific chromatin hidden Markov model (*48*) on both active and repressive histone modifications, as well as measures of physical genome compaction and nucleosome dynamics, such as sonication resistance-sequencing, MNase-seq, and H2B histone turnover (fig. S12E). We find that wild-type SOX2 efficiently binds and samples promoters as well as naïve chromatin, but not H3K9me3 or H3K27me3 (fig. S12F). Relative to SOX2, the ntTCF1-SOX2 hybrid more comprehensively bound and sampled motifs marked by H3K27me3 and moderate H3K9me3 (fig. S12G). In contrast to both SOX2 and ntTCF1-SOX2, the SOX2-ctESRRB hybrid had markedly enhanced binding at high H3K27me3 and H3K9me3 sites but at the consequence of lost ability to bind naïve chromatin (fig. S12F). In sum, non-DBD domains tune pioneer factor stability at potential motifs across the genome in different chromatin states and influence if a motif will be sampled, bound, or not sampled.

### Hybrid pioneer factors improve pluripotency reprogramming

We assessed how the expanded hybrid-stabilized binding relates to SOX2 function in its normal developmental context by comparing the newly gained sites to SOX2 binding in embryonic stem cells (ESCs). ntTCF1-SOX2 and SOX2-ctESRRB expressed in fibroblasts target 2-fold and 2.25-fold more ESC SOX2 sites compared to wild-type SOX2, respectively (fig. S13A; ntTCF1-SOX2: 1971 gained, 1761 shared, 165 lost; SOX2-ctESRRB: 2475 gained, 1543 shared, 434 lost). Gene ontology analysis (*49*) shows that, in addition to the embryonic gene program targeted by wild-type SOX2, each hybrid targets distinct additional gene sets (Fig. 4, F and G, and fig. S13, B and C). ntTCF1-SOX2 predominantly targets metabolic genes (Fig. 4F) that enable ES cells’ high multiplicative rate (*50*, *51*), whereas SOX2-ctESRRB broadly targets developmental genes (Fig. 4G, top) and not the metabolic gene set (Fig. 4G, right bottom). Our results demonstrate that non-DBD protein domains, by stabilizing binding across different SOX2 motifs, route SOX2 hybrids to target distinct genetic programs.

H3K9me3-marked heterochromatin impedes initial access to embryonic genes by pluripotency factors OCT4, SOX2, KLF4, and c-MYC (OSKM) in cellular reprogramming (*32*, *52*). While any given transcription factor binding event may not lead to productive chromatin changes, access of OSKM to the pluripotency network is required to establish and maintain a pluripotency state (*53*). As the hybrid SOX2 proteins expand chromatin targeting over both developmental and metabolic gene programs, we tested the hybrid SOX2 proteins in induced pluripotent stem cell (iPSC) reprogramming assays (fig. S14A). We transduced mouse embryonic fibroblasts (MEFs) with lentiviral O + K + M and SOX2 or ntTCF1-SOX2 or ctESRRB-SOX2, verifying robust expression at 48 hours (fig. S14B). By day 9, there was a 63% increase in alkaline phosphatase–positive pluripotent cells and colonies in the ntTCF1-SOX2 conditions and a 61% decrease in colonies the SOX2-ctESRRB conditions, compared to the SOX2 control (Fig. 4, H and I). Withdrawal of doxycycline at day 9 through 14 led to maintenance of ESC colonies in the ntTCF1-SOX2 and SOX2 conditions (Fig. 4J). By contrast, the SOX2-ESRRBct–generated colonies did not persist (fig. S14C), and immunostaining of earlier-stage cells for SOX2 showed that the partially reprogrammed colonies were devoid of ctESRRB-SOX2 expression, suggesting that negative selection may act on the hybrid factor that draws SOX2 away from its normal target sites (fig. S14, D to F) and in contrast to the ntTCF1-SOX2 that simply expands SOX2 targeting.

## DISCUSSION

Determining how developmental transcription factors engage repressive chromatin is essential for understanding how genetic programs for alternative cell fates are activated. Here, we systematically compared 13 lineage-determining transcription factors for their ability to target different repressed chromatin states, including DNase-resistant, naïve, or low-signal chromatin enriched for linker histone but not histone marks (*20*) and H3K9me3- and H3K27-marked heterochromatin. Using our previous characterization of nucleosome turnover levels across the genome (*4*), we discerned that factors target silent chromatin regions spanning a range of nucleosome dynamics. Strong nucleosome-binding pioneer factors FOXA1, PAX7, PAX3, and SOX2 target their motifs at low-turnover nucleosomes in naïve chromatin, as well as at small windows in heterochromatin that are locally depleted for the repressive modifications. GATA4 and TCF-1 engage naïve chromatin as well as moderately enriched heterochromatin. In contrast, EBF1, ESRRB, and EOMES target sites in highly enriched heterochromatin that are characterized by more dynamic, high-turnover nucleosomes, whereas ASCL1 engages highly enriched heterochromatin at sites of low-turnover nucleosomes. Together, these results reveal that pioneer factors exhibit a range of repressed chromatin targeting. The abilities may reflect their developmental and regenerative capacities: For example, ASCL1’s ability to access both naïve and heterochromatic sites may underlie its distinct capacity to reprogram fibroblast into neurons as a single transcription factor (*54*), whereas SOX2’s exclusion from H3K9me3-marked regions is critical for its role in neural development, where embryonic gene targets acquire H3K9me3 to ensure their silencing (*55*). Given the action of transcription factors in virtually all known cell fate transitions, we propose that the differential abilities to bind naive- and heterochromatin-silenced gene targets enable groups of pioneer factors to coordinately overcome the diverse chromatin barriers that normally maintain cell identity and hence allow them to change cell fate.

Transcription factors function across a finely tuned window of protein expression, and differences in concentration may influence the chromatin targeting properties of the factor (*56*, *57*). Local concentration gradients achieved through nuclear compartmentalization and transcription factor multimerization, together with the preexisting chromatin state, further modulate the ability to recruit chromatin remodelers and generate chromatin accessibility (*45*, *58*–*60*). The sensitivity to dosage has complicated efforts to define the inherent properties of individual factors, let alone compare developmentally distinct ones. To address this, we ectopically expressed transcription factors at comparable protein concentrations within their normal physiological range, enabling a direct comparison of their chromatin targeting in a single epigenetic background. At comparable protein expression, we observe marked differences in transcription factors’ ability to target DNase-resistant, low H2B–turnover chromatin, a prerequisite to initiate chromatin opening. Pioneer factors such as FOXA1, PAX7, and PAX3 (*17*, *34*) target 20- to 30-fold more low-turnover nucleosomal sites than the nonpioneers HNF4A and RUNX1 (*27*, *61*, *62*), consistent with pioneer factors driving cell fate changes, while nonpioneers partner with other proteins and/or uphold homeostatic genetic networks.

In nontransformed primary fibroblasts, transcription factors with structurally conserved DBDs show similar nucleosome targeting properties, extending in vitro findings that different DBD types recognize DNA motifs at different positions on nucleosome surface (*7*, *9*). Recent studies have established that transcription factors target nucleosomes along a continuum of affinities (*63*), and likewise, our results place pioneer and nonpioneer factors toward different ends of this spectrum. We therefore propose that, although subtle chromatin targeting behaviors may vary between cell types, intrinsic differences in factor ability to engage distinct chromatin states provide flexibility of transcription factors to combinatorially elicit cell fate changes.

On the basis of findings that non-DBD regions shape pioneer factor target choice (*4*, *13*, *42*, *43*, *46*, *64*), we designed SOX2 hybrid proteins with both structured and intrinsically disordered non-DBDs from factors that better target densely marked heterochromatin. Notably, the SOX2 hybrids nonredundantly shifted SOX2’s chromatin targeting preferences without profoundly changing motif preference, demonstrating that the domains facilitate the chromatin search and engagement process rather than specific binding. Our SOX2-ctESRRB fusion shifted engagement to H3K9me3 and H3K27me3 sites at the expense of weaker targeting to naïve chromatin, while the ntTCF1-SOX2 fusion retained naïve targeting and expanded moderate heterochromatin targeting. During pluripotency reprogramming, the failure of SOX2-ctESRRB to engage naïve sites was associated with a failure to generate iPSCs, while the expanded targeting of ntTCF1-SOX2 associated with improve iPSC formation. We conclude that the non-DBD domains confer functional access to different closed chromatin states and are not generic. Further understanding of how these domains engage specific chromatin states will facilitate engineering of transcription factors that activate specific genetic networks and reprogram new cell fates. Given the limited hybrid swaps tested here, and emerging studies finding extensive cooperative interactions between transcription factors (*65*, *66*), we anticipate that there exists in nature a highly diverse set of nonredundant non-DBDs that collectively shape transcription factor binding during cell fate control.

## MATERIALS AND METHODS

### Plasmid construction

Coding regions for expression vectors were determined from UniProt and synthesized [Integrated DNA Technologies (IDT)], fused to a V5 epitope, separated by a GSGSTS flexible linker. Sequences were designed with 20-bp homologous overhangs to a TetO-FUW lentiviral expression vector digested with EcoRI (TetO-FUW-OCT4, Addgene, #20323) (table S2). Coding sequences were inserted into a TetO-FUW vector using NEB Assembly 2× Master Mix (New England Biolabs, E2611), according to the manufacturer’s instructions.

### Cell culture

Human BJ foreskin fibroblasts and human embryonic kidney 293T cells were obtained from American Type Culture Collection (BJ: CRL-2522, 293T: CRL-3216) and cultured in Eagle’s Minimum Essential Medium (EMEM; Sigma-Aldrich, M2279) supplemented with 10% fetal bovine serum (FBS; HyClone, SH30071) and 2 mM GlutaMAX (Thermo Fisher Scientific, 35050061) at 37°C and 5% CO_2_.

MEFs for iPSC reprogramming were cultured in EMEM in supplemented with 10% FBS, 2 mM GlutaMAX, nonessential amino acids (1:100; Gibco, 111404050) and 1 μM β-mercaptoethanol. iPSCs were cultured in MEF media supplemented with 1000 IU of recombinant mouse leukemia inhibitory factor (LIF; Sigma-Aldrich, ESG1107).

### Lentiviral production

Approximately 6 × 10^6^ cells were plated in 15-cm culture dishes and incubated overnight at 37°C. After 24 hours, the cells were transfected using 9 μg of pMD2.G (Addgene, #12259), 13.5 μg of psPAX2 (Addgene, #12260), and 18 μg of lentiviral expression plasmid. The following day, media was freshly changed. The supernatant containing unconcentrated lentivirus was collect at 24, 48, and 72 hours following media change. Lentivirus was concentrated by mixing 1:4 with concentrator solution (40% polyethylene glycol and 1.2 M NaCl), incubating overnight, and centrifuging at 1600*g* for 30 min at 4°C. Precipitated virus was resuspended 1:100 in phosphate-buffered saline (PBS).

### Western blotting

Nuclear protein lysates were harvested as in (*67*), and 10 μg of protein was run on a NuPAGE 4 to 12% bis-tris gel (Thermo Fisher Scientific, NP0323PK2). Western blot was then performed according to a conventional protocol, with membranes incubated with primary antibody V5 (1:1000; Invitrogen, R960-25), TATA Binding Protein TBP (1:1000; Abcam, ab63766), and pol II (1:1000; Abcam, ab140509), and a horseradish peroxidase–conjugated anti-mouse (1:15,000; Invitrogen, 31430), anti-rabbit (1:15,000; Invitrogen, 31460), or anti-goat secondary (1:15,000; Invitrogen, 31402). Detection was performed with ECL Prime reagent (SuperSignal West Pico PLUS Chemiluminescent Substrate, Thermo Fisher Scientific, 34580) and an Amersham 600 imager.

### Immunostaining

Fibroblasts were fixed in 4% paraformaldehyde for 20 min at room temperature, washed twice for 5 min with room temperature PBS, permeabilized with chilled PBS + 0.1% Triton in PBS for 10 min, and briefly washed twice with PBS + 0.1% Tween (PBST). Fixed fibroblasts were then blocked with 3% donkey serum in PBST for 1 hour at room temperature. Primary V5 (1:500; Invitrogen, R960-25), OCT4 (1:100; Santa Cruz Biotechnology, C-10), KLF4 (1:100; R&D Systems, AF3640), c-MYC [1:100; Cell Signaling Technology (CST), D84C12], and SOX2 (1:500; R&D Systems, AF2018) antibody staining in blocking buffer was performed at 4°C overnight. After primary incubation, the fibroblasts were washed three times for 15 min with PBST and incubated with a donkey-anti-mouse, donkey-anti-rabbit, or donkey-anti-goat Alexa Fluor–conjugated 488 or 564 secondary antibody for 1 hour. The fibroblasts were than washed three times for 15 min with PBST, counterstained with 4′,6-diamidino-2-phenylindole (DAPI), washed a final time with PBS, and imaged on a wide-field fluorescent microscope (Nikon, TE2000-U).

We quantified the fluorescence signal of antibody staining in FIJI v2.14.0 (*68*) by first identifying nuclei from the DAPI channel as regions of interest (ROIs) by executing a macro with commands: run(“Threshold...,” “lower=50 upper=250 stack”); run(“Analyze Particles...,” “size=200-Infinity circularity=0.00-1.00 show=Masks display clear add”); nROIs = nResults. Next, antibody staining images corresponding to the nuclei ROIs were opened, all ROIs were selected, and fluorescent signal was measured with the Analyze/Measure command. Images used for quantification are in the additional supplementary figure below.

### Chromatin immunoprecipitation sequencing

In biological duplicates, ChIP-seq of transcription factors was performed as in (*4*). Briefly, 1 × 10^6^ human BJ fibroblasts were seeded onto two 15-cm-diameter tissue culture plates. Once attached, the fibroblasts were transduced with 5 ml of unconcentrated rTTA2 lentivirus, transcription factor-encoding lentivirus at a multiplicity of infection (MOI) of 1.25, in a total volume of 20-ml culture media supplemented with polybrene (8 μg/ml). Eighteen hours after lentiviral transduction, the cells were washed twice with PBS, and ectopic transcription factors were induced with 1 μg/ml. After 48 hours of expression, 1% formaldehyde was added directly to tissue culture dishes, which were then rocked for 10 min, and quenched with 125 mM glycine. Culture dishes were washed three times with ice-cold PBS, and fibroblasts were scraped off plates and pelleted. Cell pellets were then freeze thawed three times on dry ice, followed by resuspension in 5 ml of ice-cold hypotonic buffer [20 mM Hepes-KOH (pH 7.5), 20 mM KCl, 1 mM EDTA, 10% glycerol, 1 mM dithiothreitol (DTT), and complete protease inhibitors cocktail] and incubated on a wheel for 10 min at 4°C. After a 5-min centrifugation at 2000 rpm, the pellet was resuspended in 5 ml of ice-cold lysis buffer [50 mM Hepes-KOH (pH 7.5), 140 mM NaCl, 1 mM EDTA, 10% glycerol, 0.5% NP-40, 0.25% Triton X-100, 1 mM DTT, and complete protease inhibitors cocktail] and incubated on a wheel for 10 min at 4°C. The cells were then dounced five times to isolate the nuclei and centrifuged 5 min at 2000 rpm. The pellet was resuspended in 10 ml of ice-cold wash buffer (buffer III) [10 mM tris-HCl (pH 8), 200 mM NaCl, 1 mM EDTA, 0.5 mM EGTA, 1 mM DTT, and complete protease inhibitors cocktail] and incubated on a wheel for 10 min at 4°C. After 5 min of centrifugation at 2000 rpm, the pellet of nuclei was frozen on dry ice, before thawing and resuspension in 2 ml of ice-cold sonicatioqysis buffer (buffer IV) [10 mM tris-HCl (pH 8), 100 mM NaCl, 1 mM EDTA, 0.5 mM EGTA, 0.1% Na-deoxycholate, 0.5% *N*-lauroylsarcosine, 1 mM DTT, and complete protease inhibitors cocktail]. The nuclei were then sonicated for 10 min on a COVARIS sonicator, leading to fragment sizes around 300 to 500 bp. Before the immunoprecipitation step, the chromatin extracts were solubilized with 1% Triton and rocked at least 10 min, and the supernatant was saved after centrifugation at the maximum speed for 20 min. For each replicate, 25 μg of chromatin was incubated with 1 μl of V5 antibody (Invitrogen, R920-25) and rocked overnight at 4°C. Before adding the antibody, 100 μl total was uptake as inputs for each condition. The next day, 50 μl of Dynabeads Protein G (Thermo Fisher Scientific, catalog no. 10003D) saturated overnight with bovine serum albumin (1 mg/ml) in sonication buffer was added to the antibody/chromatin mix and rocked for 3 hours at 4°C. The beads were washed for a total of five times with the ice-cold ChIP washing buffer (50 mM Hepes-KOH, 500 mM LiCl, 1 mM EDTA, 1% NP-40, and 0.7% NaDOC + Complete EDTA-free from Roche) and once with tris-EDTA. The beads and the inputs were then resuspended in 150 μl of ChIP elution buffer (1% SDS and 0.1 M NaHCO_3_) and decrosslinked overnight at 65°C. The next day, 150 μl of tris-EDTA was added to each tube, with 5 μl of ribonuclease A (10 mg/ml) and incubated for 2 hours at 37°C. Five microliters of Proteinase K (10 mg/ml) was then added to the mix and incubated for 1 hour at 55°C. DNA was then purified and concentrated (Zymo Research, ChIP DNA Clean and Concentrator, D5205). Libraries were made using NEBNext Ultra II for DNA Library Prep (New England Biolabs, E7645) and paired-end sequenced on an Illumina NextSeq1000.

### ChIP-seq analysis

Paired-end reads were aligned to human genome hg19 using bowtie2 (v2.3.4.3) (*69*) with run parameters --local --X 1000. A quality-filtered bam file was generated with the command samtools view -q 5 -bS (SAMtools v1.1) (*70*). Optical and polymerase chain reaction duplicate reads were marked and removed using PICARD MarkDuplicates REMOVE_DUPLICATES = TRUE ASSUME_SORT_ORDER = queryname (GATK 4.2.6.0) (*71*). BED files were generated using bedtools (v2.26.0) (*72*) bamToBed. Coverage maps of IP and input samples were calculated using bedtools genomeCoverageBed, reads per million (RPM) normalized, and output in a bedGraph format. The IP sample was subtracted by the input sample, and bedGraph files were processed to bigwigs using UCSC bigWigAverageOverBed (v2) (*73*).

ChIP-seq peaks were called using MACS2 (v2.2.7.1) (*74*) callpeak with options -f BEDPE. Peaks detected in all biological replicates were combined to form a consensus peak set, as analyzed in Figs. 1 and 2. For Figs. 3 and 4, comparing wild-type and SOX2 hybrid factors or SOX2 mutants, peaks were classified as “shared” if detected in at least one replicate of either condition and as “unique” if detected in biological replicates for one condition but absent from all replicates of the other.

Preexisting DNase accessibility signal underlying transcription factor peaks was determined using bigWigAverageOverBed (v2). Peaks with a DNase value greater than 0.05 were annotated as accessible chromatin and, otherwise, were annotated as inaccessible chromatin, using command “awk ‘$4 < 0.05’ TF_with_DNase.bed > TF_resistant.bed; awk ‘$4 > 0.05’ TF_with_DNase.bed > TF_accessible.bed.” DNase cutoff was determined by empirical testing.

### Motif analysis

Motif analysis on peak sets was performed with HOMER-v4.6 (*75*) using findMotifsGenome.pl, with de novo motifs reported in fig. S2A. To map the distribution of motifs in different chromatin states, we identified motif instances across the genome using the position weight matrices from fig. S2A using scanMotifGenomeWide.pl, identified the DNase accessibility across the central 100 bases using bigWigAverageOverBed --sampleAroundCenter=100, and then split accessible motifs or inaccessible motifs by the 0.05 RPM threshold.

To determine differential motif enrichment across DNase accessibility bins, we used monaLisa v1.6.0 (*29*). Briefly, TF peak files were annotated with DNase I hypersensitivity using bigWigAverageOverBed -bedOut=TF_with_DNase.bed and read into R with BSgenome v1.4.3. Peaks were grouped into 10 equal sized bins according to DNase levels. Peak widths were standardized to mean size and recentered on the center with GenomicRanges v1.52.1, and genomic sequences were extracted from the hg19 reference genome using Biostrings v2.68.1. Motif position weight matrices for vertebrate transcription factors were retrieved from the JASPAR2020 database via TFBSTools v1.38.0. Motif enrichment across bins was calculated using calcBinnedMotifEnrR(). Motifs were considered as significant and differentially enriched across bins if they passed a cutoff of −log_10_adj(*P* > 2) and a foreground frequency of >5%. Enriched motifs were visualized using ComplexHeatmaps, displaying log_2_ enrichment between bins.

### Motif sampling

To identify the chromatin sampling of SOX2, we used genome-wide instances of the de novo SOX motif (fig. S2A). Motifs were classified as bound or not with SOX2 peaks by bedtools intersect -v. To order motifs in heatmaps, mean transcription factor ChIP-seq enrichment across central 100 bases of motifs was calculated using bigWigAverageOverBed --sampleAroundCenter=100 and then sorted from high to low. To exclude low-coverage and unmappable regions of the genome from the sampling analysis, we filtered out motifs in which there was >75% of the region covered by at least one read (“covered,” column 3, in bigWigAverageOverBed output). We then generated backgrounds with the motif file shifted +10 kB in unix with the command: awk ‘BEGIN(OFS=“\t”) {\($2 += 10000; $3 += 10000; print)’ motif.bed > 10kB_shifted_motif.bed. Ordered heatmaps and background motif sets were then used to generate heatmaps.

We generated a 10-state chromHMM model as described in (*48*). Briefly, we compiled binarized BEDs for H3K4me3 (ENCFF394CAJ), H3K36me3 (ENCFF189UOB), H3K9me3 (ENCSR624YTO), H3K27me3 (ENCSR000DQG) from ENCODE (*76*), 6h H2B turnover, high and low MNase (*4*), euchromatin-sequencing and sonication resistance heterochromatin-sequencing (*33*), and H1 CUT&RUN (this study). We then trained a 10-state chromHMM model with command java -mx1600M -jar ChromHMM.jar LearnModel -p 8 10_state_model_hBJ 10 hg19. On the basis of emission features as well as manual assessment on a genome browser, we annotated the 10-state model with genomic features associated with the chromatin states. We calculated genomic enrichment of bound, sampled, or unsampled motifs of these states over SOX2, hybrid factors using command java -mx1200M -jar ChromHMM.jar OverlapEnrichment -colfields 0,1,2,3 -labels 10_state_model_hBJ.bed SOX2_motif_classX.bed SOX2_motif_classX_state_enrichment.txt.

### Heatmap plots

Heatmaps were generated using computematrix reference-point (deeptools v3.5.2), with options --skipZeros and --missingDataAsZero, and with heatmap window designated as presented in figures. Heatmap count matrices were plotted using plotHeatmap (deeptools v3.5.2), with columns sorted by chromatin state and not transcription factor, for example, sorting DNase I, 6h H2b, H3K9me3, and H3K27me3. Metaplots were generated with the plotProfile command (deeptools v3.5.2).

### Gene ontology of transcription factor targets

To identify pathways associated with wild-type or hybrid SOX2 targeting, we used the Genomic Regions Enrichment of Annotations Tool (*43*) web tool on the different ChIP-seq binding site sets. We use the two nearest genes to transcription factor binding site option, set with a 50-kb window. The top five most significant terms from each ontology analysis were plotted in Fig. 4 and fig. S13, with all identified genes in table S3.

### iPSC reprogramming

A total of 50,000 MEFs on passage 2 or 3 were seeded in a 12-well dish and incubated overnight. The following day, MEFs were transduced at an MOI of 5 with rTTA-tetO lentivirus and individual OCT4 + KLF4 + cMYC and SOX2-V5 or ntTCF1-SOX2-V5 or V5-SOX2-ctESRRB lentivirus with MEF media supplemented with polybrene (8 μg/ml). Eighteen hours after lentiviral transduction, the cells were washed twice with PBS, and reprogramming factors were induced with doxycycline (1 μg/ml). Dox-containing media was subsequently changed every 48 hours. On day 2, two biological replicates were harvested for Western blotting and immunostaining. After 3 days of induction, MEF media was supplemented with 1000 IU of ESGRO LIF (Sigma-Aldrich, ESG1107). After LIF addition, half the media in each well was changed daily. On day 9 of reprogramming, biological triplicates were fixed and stained for alkaline phosphatase, following the manufacturer’s instructions (Sigma-Aldrich, MAK447). Stained colonies were counted in FIJI using the Cell Counter program (*68*). Additional biological replicates were withdrawn from doxycycline from days 9 to 14 to measure colony maintenance in the absence of transgene expression. Daily images were taken by wide-field light microscopy.
