# Supplementary material for: Basis for lineage-determining pioneer factors targeting distinct repressed chromatin states
Source: Sci Adv. 2026 Jan 9;12(2):eadz7409. doi: 10.1126/sciadv.adz7409 (PMC12787584; doi:10.1126/sciadv.adz7409)
Supplement: Supplementary file 1 — Figs. S1 to S14 Tables S1 and S2 Legend for table S3 [file sciadv.adz7409_sm.pdf]

Science Advances
AAAS

Supplementary Materials for

Basis for lineage-determining pioneer factors targeting distinct repressed chromatin states

Andrew Katznelson *et al.*

Corresponding author: Kenneth S. Zaret, zaret@pennmedicine.upenn.edu

Sci. Adv. **12**, eadz7409 (2026)
DOI: 10.1126/sciadv.adz7409

The PDF file includes:

Figs. S1 to S14
Tables S1 and S2
Legend for table S3

Other Supplementary Material for this manuscript includes the following:

Table S3

Katznelson et al., Supplemental Figure 1

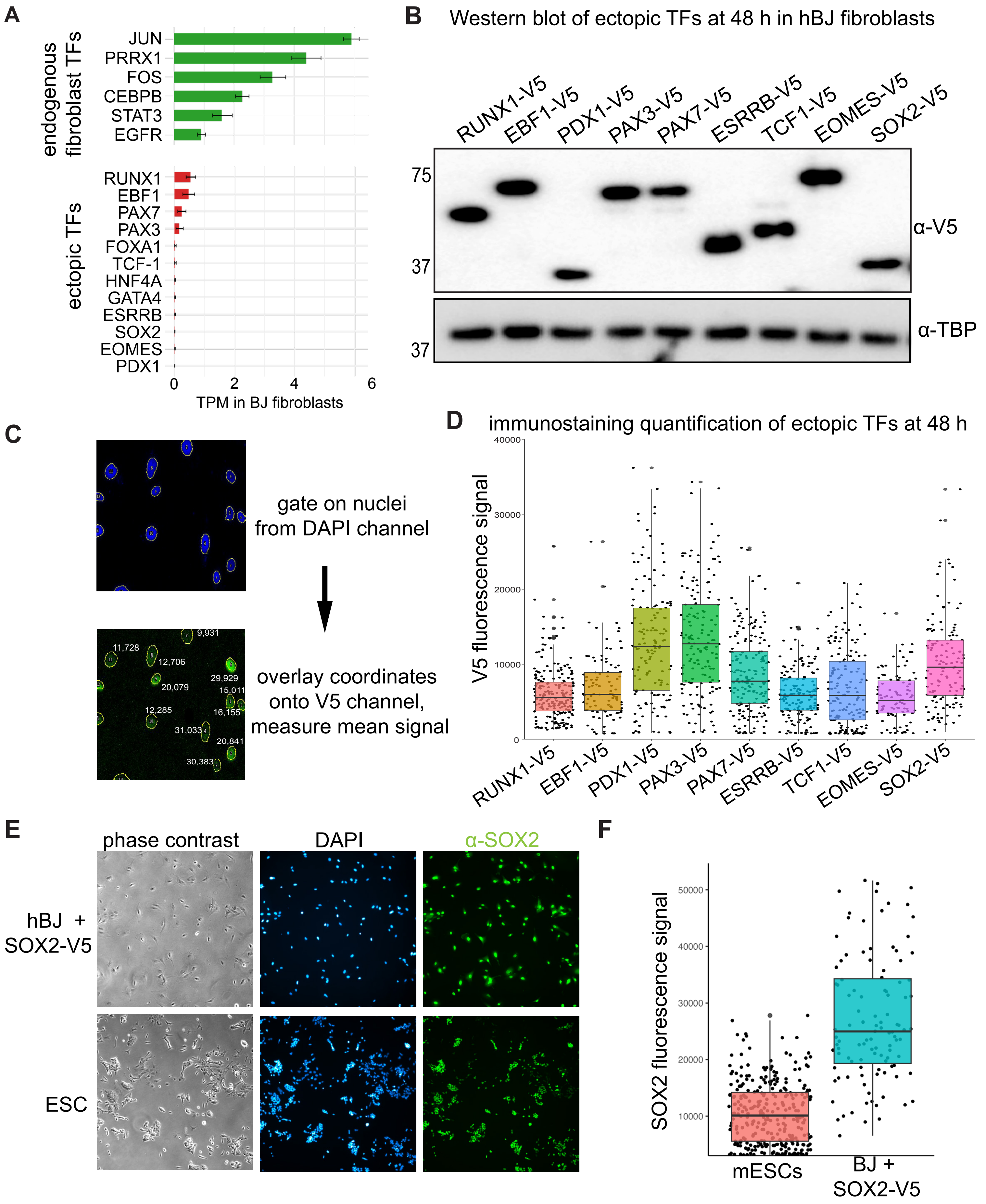

Fig. S1. Ectopic expression of developmentally diverse transcription factors. (A) BJ fibroblast RNA-seq TPM values (*76*) of endogenous fibroblast transcription factors vs. ectopic transcription factors. **(B)** Western blot of ectopic transcription factors after 48 h in BJ fibroblasts. **(C)** Analysis schematic to quantify immunofluorescence signal. **(D)** Quantification of immunostaining of ectopic transcription factors at 48 hours in BJ fibroblasts. **(E)** Phase contrast and immunostaining with SOX2 antibody of ectopic fibroblast SOX2-V5 and SOX2 in ESCs. **(F)** Quantification of SOX2 immunostaining from panel E.

Katznelson et al., Supplemental Figure 2

A

de novo motif analysis of 48 h ChIP-seq peaks

TF	de novo PWM	closest known motif	p-val
ASCL1	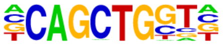	(bHLH) Tfap4	1e-9194
EBF1	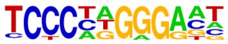	proBcell-EBF	1e-8795
EOMES	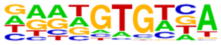	Tbet-Tbox	1e-482
ESRRB	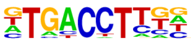	Esrrb(NR)	1e-2609
FOXA1*	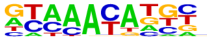	Forkhead_class	1e-3124
GATA4**	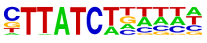	GATA2	1e-9474
HNF4A*	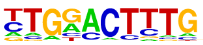	HNF4A	1e-1189
PAX3	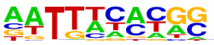	MA0075.1_Prrx2	1e-3727
PAX7	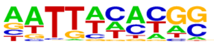	MA0075.1_Prrx2	1e-4012
PDX1	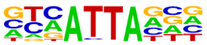	NKX6.1	1e-1124
RUNX1	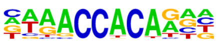	RUNX2	1e-4821
SOX2	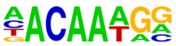	SOX3	1e-2723
TCF-1	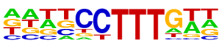	Tcf3	1e-2338

* 48 hour ectopic BJ FOXA1/HNF4A ChIP-seq re-analyzed from GSE220570
** 48 hour ectopic BJ GATA4 ChIP-seq re-analyzed from GSM2401452

B

pairwise comparison of de novo motif enrichment

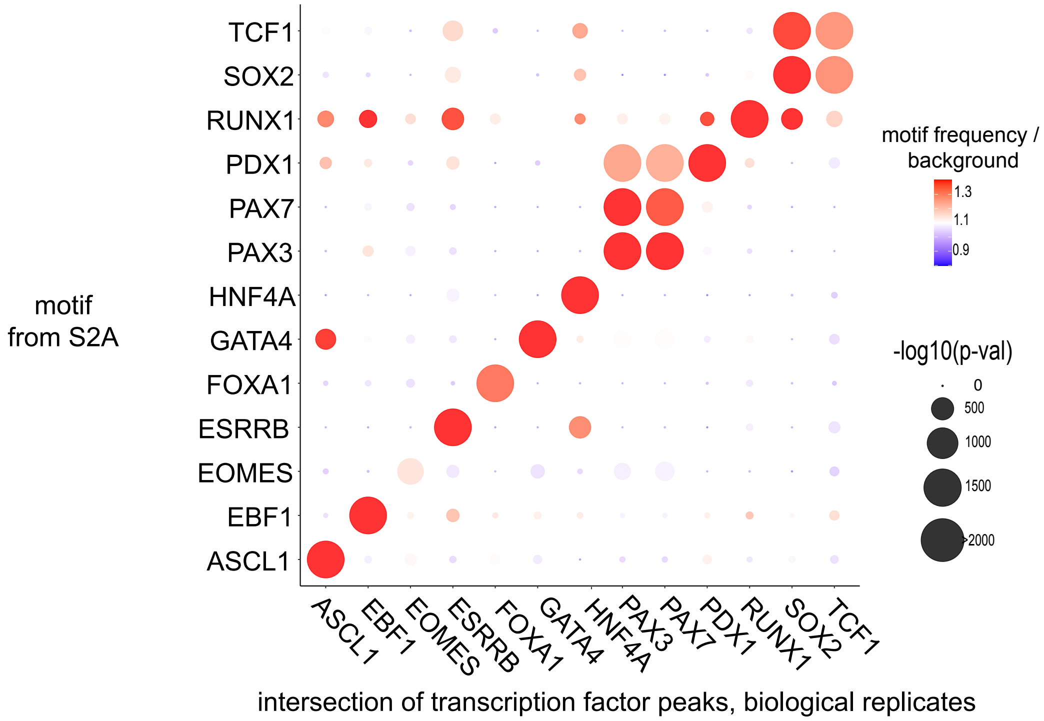

Fig. S2. Ectopic transcription factors target specific, cognate DNA motifs. **(A)** Summary of *de novo* motif calling over fibroblast transcription factor ChIP-seq peaks. Peak sets used for downstream analysis consist of intersection between biological replicates. **(B)** Pairwise motif enrichment analysis across ectopic fibroblast ChIP-seq peaks.

Katznelson et al., Supplemental Figure 3

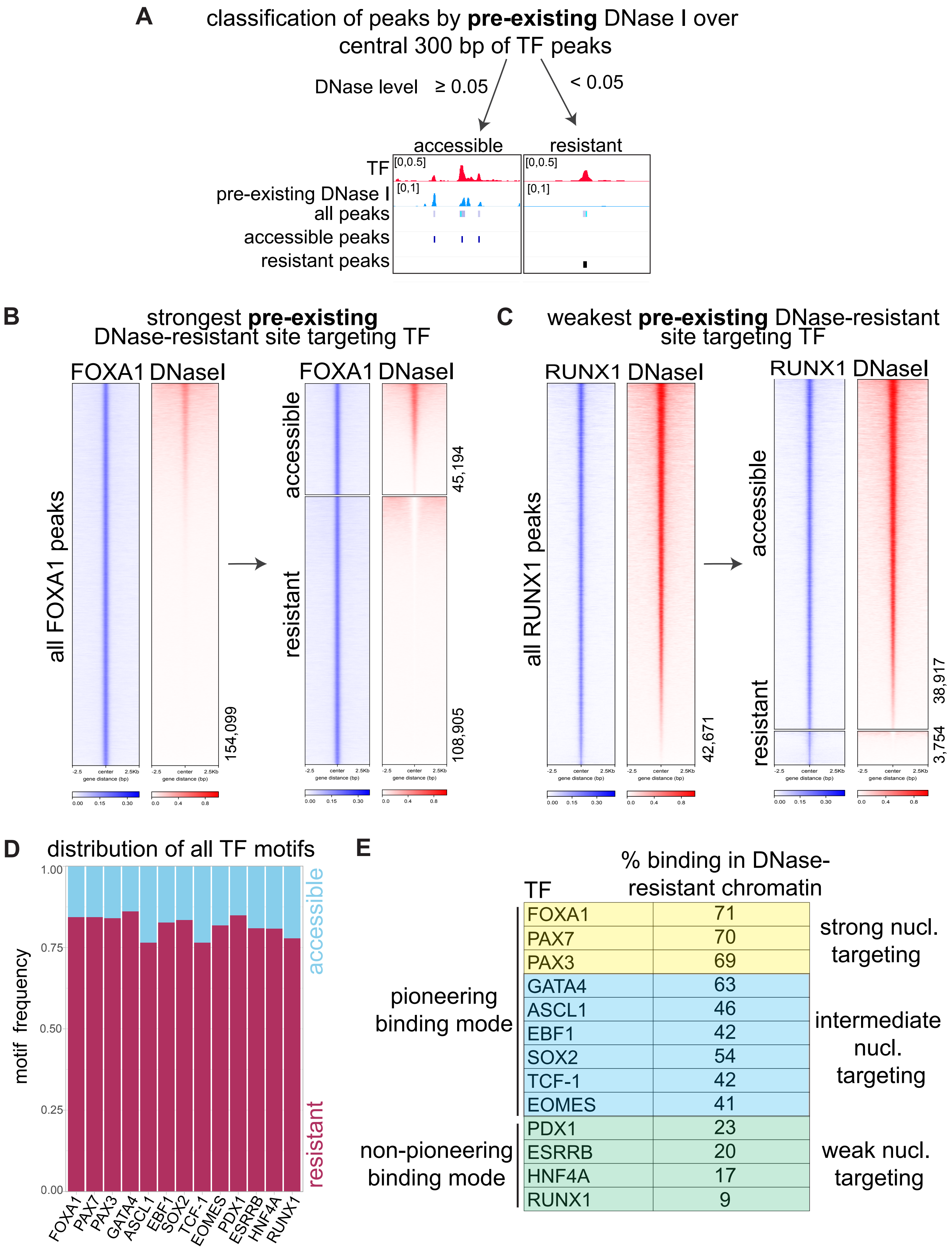

TF	% binding in DNase-resistant chromatin
FOXA1	71
PAX7	70
PAX3	69
GATA4	63
ASCL1	46
EBF1	42
SOX2	54
TCF-1	42
EOMES	41
PDX1	23
ESRRB	20
HNF4A	17
RUNX1	9

Fig. S3. Partitioning of chromatin binding by pre-existing DNase I. (A) Scheme and examples for assignment of ectopic transcription factor peaks by pre-existing DNase accessibility (open chromatin), or DNase resistance (closed chromatin). **(B-C)** Chromatin accessibility stratification of **(B)** FOXA1 and **(C)** RUNX1 binding with pre-existing DNase I signal **(D)** Genomic positions of motifs from Fig. S2A in DNase-accessible or resistant chromatin prior to transcription factor expression. **(E)** Summary table of targeting to DNase resistant sites.

Katznelson et al., Supplemental Figure 4

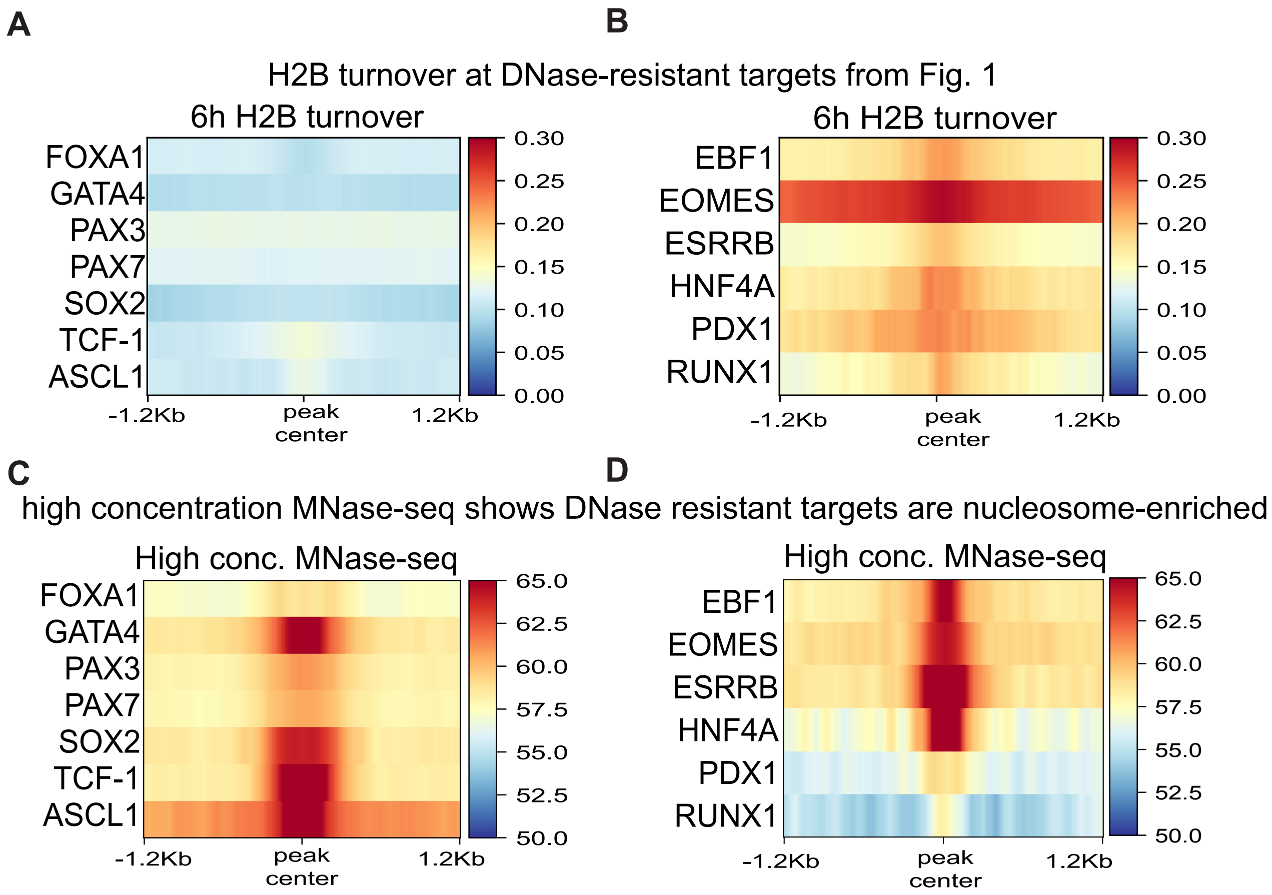

Fig. S4. Differential targeting of high- or low-histone turnover chromatin. (A-B) 6 h H2B turnover at DNase resistant sites of **(A)** pioneer factors which target stable chromatin, and **(B)** pioneer and non-pioneer factors which target high histone exchange, dynamic chromatin. **(C-D)** Confirmation of nucleosome targeting by enrichment of high concentration MNase-seq over DNase resistant targets from the pioneer factors in **(C)** Fig. S4A**,** and **(D)** Fig. S4B.

Katznelson et al., Supplemental Figure 5

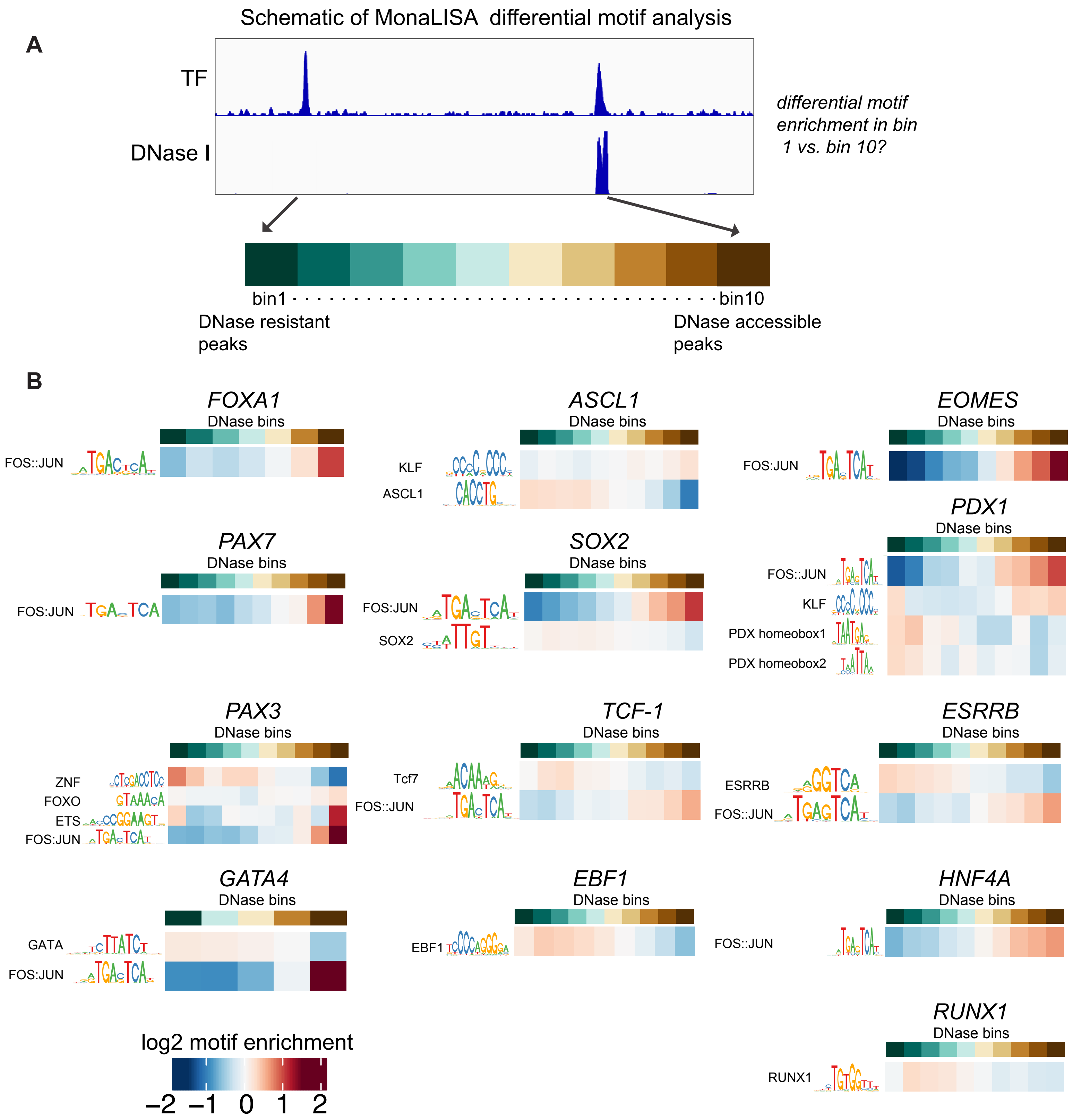

Fig. S5. Identification of motifs differentially enriched by DNase I state. (A) Schematic of MonaLisa motif analysis to identify differentially enriched motifs in DNase resistant vs. DNase accessible transcription factor binding sites. **(B)** MonaLisa analysis of ectopic transcription factor peaks binned by DNase levels, log2(motif enrichment) of differential motifs plotted.

Katznelson et al., Supplemental Figure 6

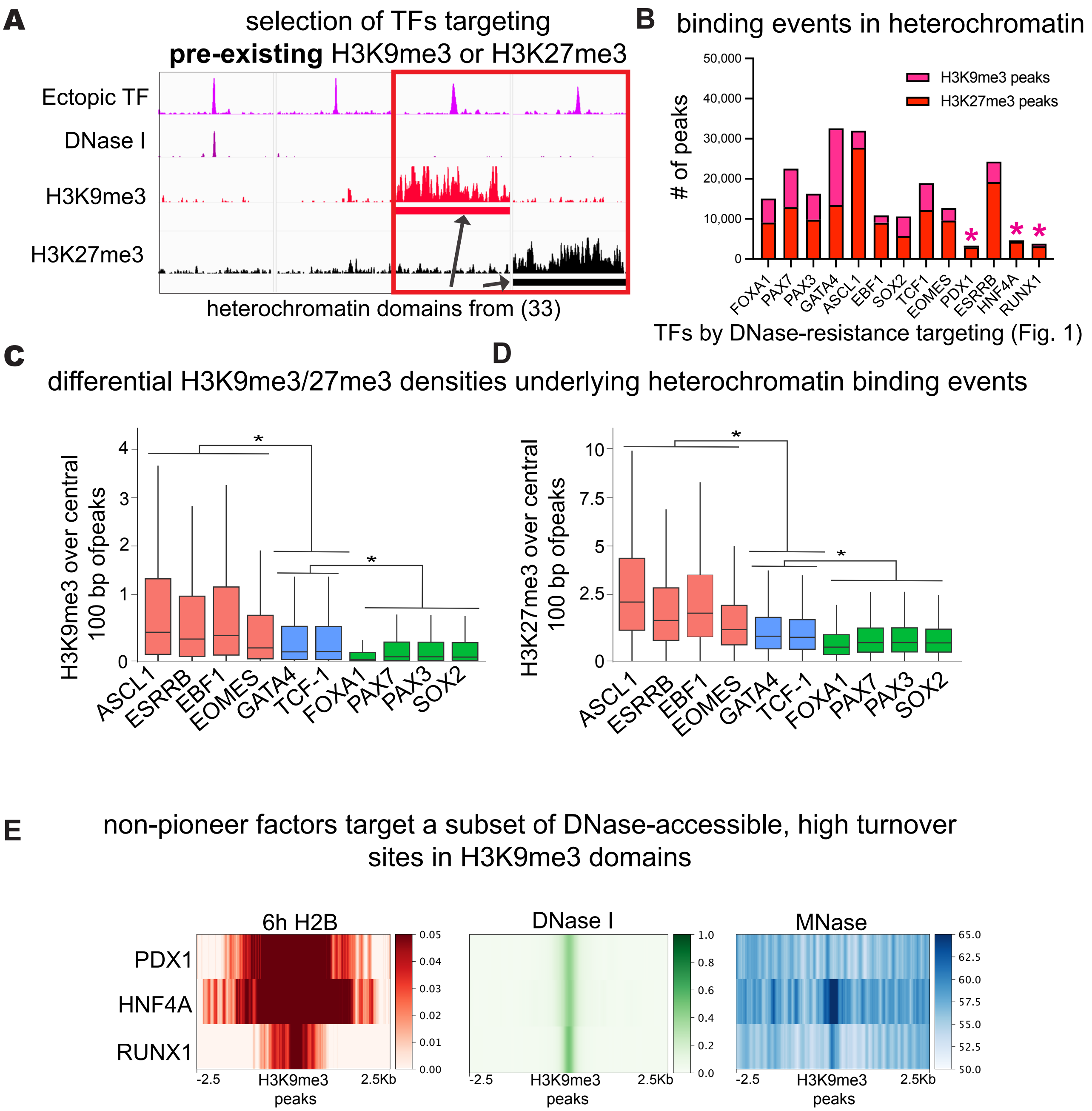

Fig. S6. Analysis of heterochromatin binding events by ectopic transcription factors. (A) Example classification of binding events overlapping sonication-resistant H3K9me3 or H3K27me3 heterochromatin domains from (*33*). **(B)** Summary bar plot of number of binding events for each transcription factor in heterochromatin. Pink asterisks denote factors with minimal heterochromatin binding. **(C-D)** Quantification of H3K9me3 and H3K27me3 underlying pioneer factor targets. * $p < 0.001$ as determined by Mann-Whitney U test. **(E)** Heatmaps of 6 h H2B, DNase I, and MNase-seq signal underlying non-pioneer H3K9me3 binding events.

Katznelson et al., Supplemental Figure 7

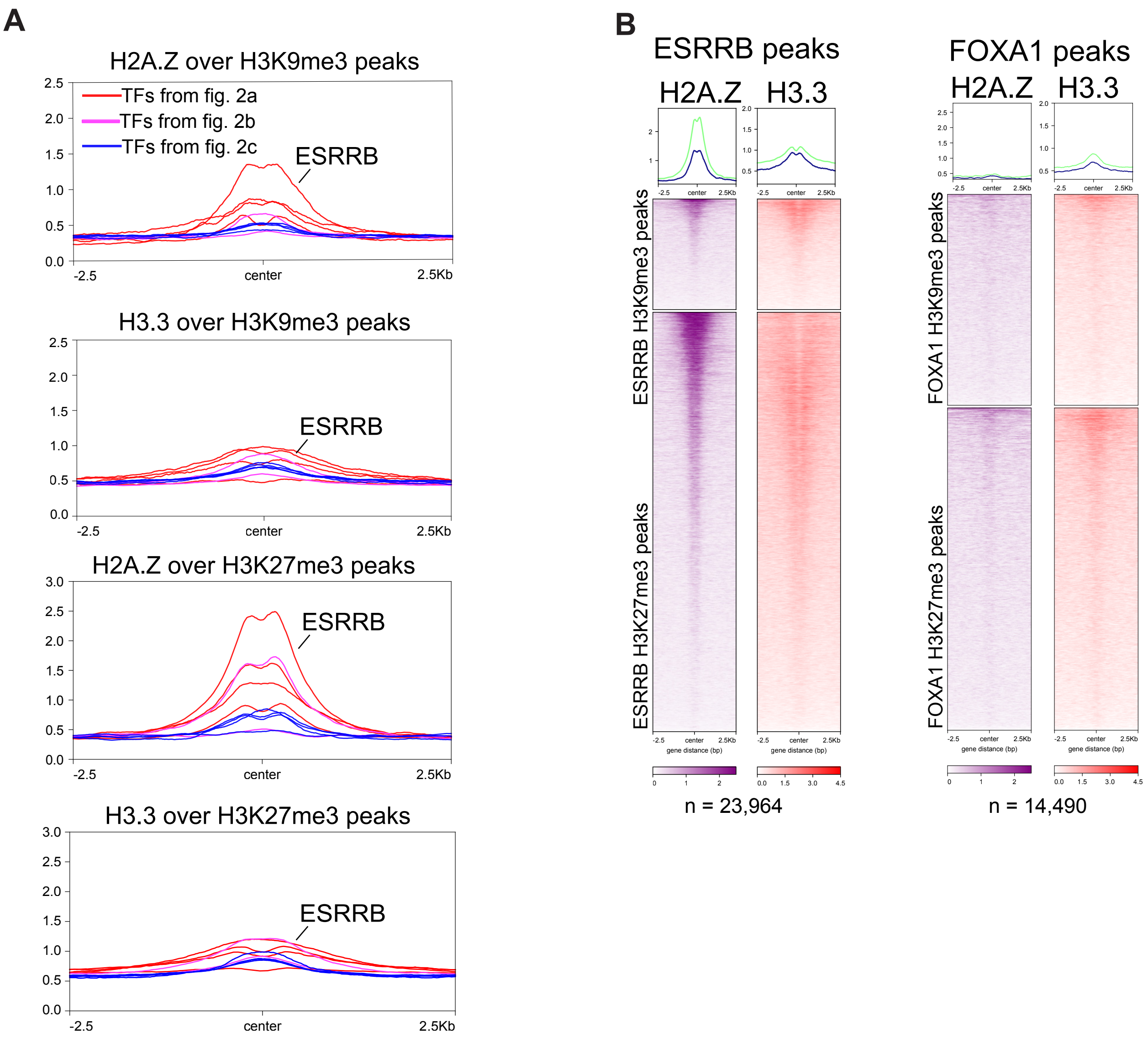

Fig. S7. Histone variant enrichment distinguishes between different heterochromatin binding modes. (A) Enrichment of pre-existing H2A.Z and H3.3 over H3K9me3 (top) and H3K27me3 (bottom) heterochromatin binding events. **(B)** Exemplar heatmap of differential H2A.Z and H3.3 across heterochromatin binding events for ESRRB (left) and FOXA1 (right).

Katznelson et al., Supplemental Figure 8

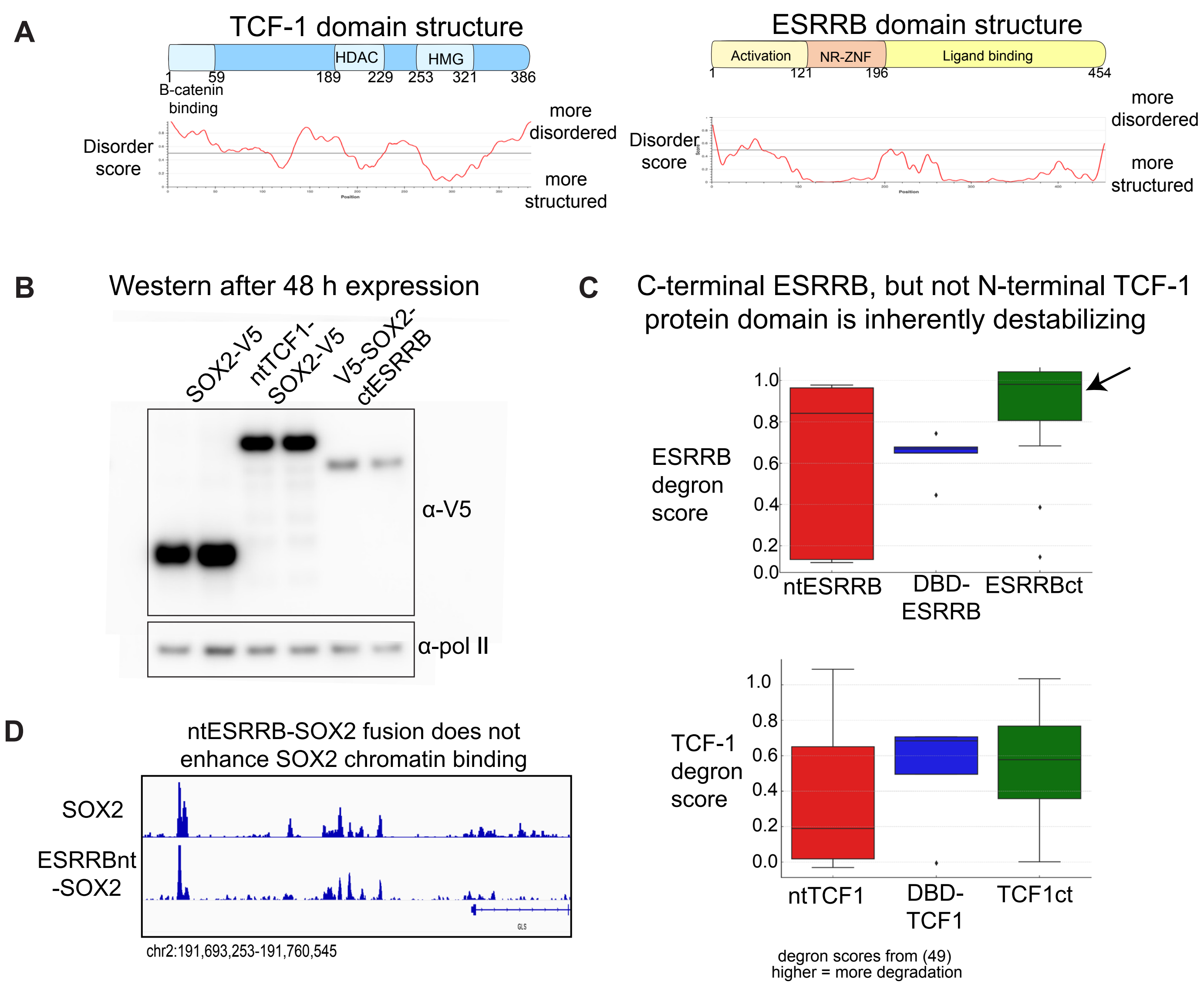

Fig. S8. Ectopic expression of hybrid ntTCF1-SOX2 and SOX2-ctESRRB. (A) TCF-1 and ESRRB protein domain structure with predicted protein disordered (IUpred2A, bottom). **(B)** Western blot of SOX2-V5, ntTCF1-SOX2-V5, V5-SOX2-ctESRRB after 48 h expression. **(C)** Inherent degradation capacity for different protein segments of ESRRB (left), and TCF-1 (right), with degron scores from (*47*). **(D)** Genome browser view of ntESRRB-SOX2 hybrid.

Katznelson et al., Supplemental Figure 9

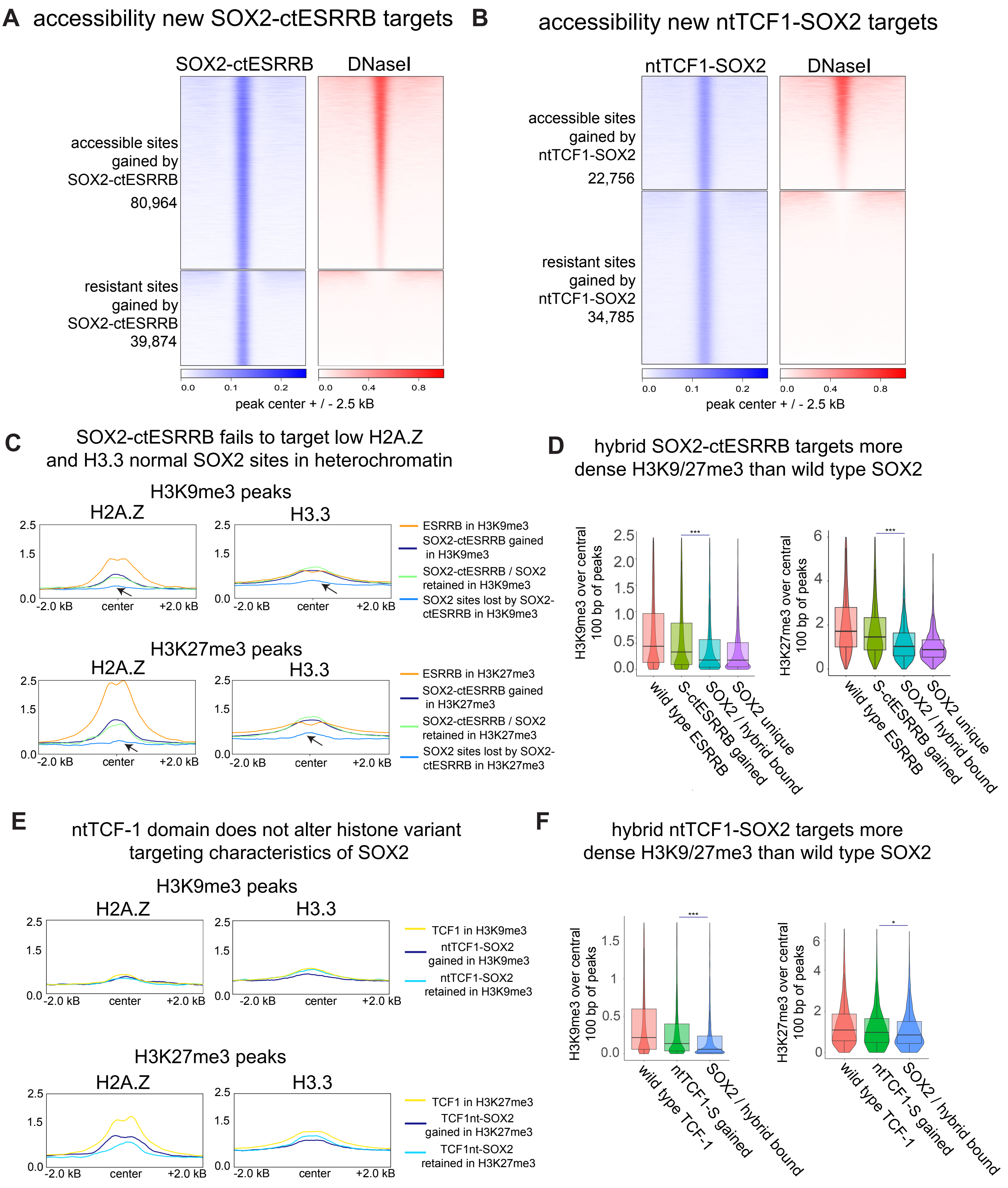

Fig. S9. Hybrid ntTCF1-SOX2 and SOX2-ctESRRB target different chromatin states. Heatmap of TF and pre-existing DNase I at sites gained vs. wild type SOX2 by **(A)** SOX2-ctESRRB, and **(B)** ntTCF1-SOX2 **(C)** H2A.Z and H3.3 enrichment over ESRRB, SOX2-ctESRRB, SOX2 heterochromatin binding sites. Arrows denote SOX2 peak set unbound by SOX2-ctESRRB. **(D)** H3K9me3, H3K27me3 enrichment over ESRRB, SOX2-ctESRRB, SOX2 heterochromatin binding sites. **(E)** H2A.Z and H3.3 enrichment over TCF-1, ntTCF1-SOX2, SOX2 heterochromatin binding sites. **(F)** H3K9me3, H3K27me3 enrichment over TCF-1, ntTCF1-SOX2, SOX2 heterochromatin binding sites. *** $p < 0.001$, * $p < 0.05$ for al panels, as determined by Mann-Whitney U test.

Katznelson et al., Supplemental Figure 10

Sox2 motif enrichment across hybrid targets

A

set	n	% in targets or background 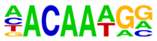		p-val
SOX2 unique	2,778	47.55%	32.03%	1e-64
SOX2 / ntTCF1-S shared	81,347	50.53%	30.75%	1e-2509
SOX2 / ntTCF1-S unique	57,541	41.27%	29.43%	1e-792

B

set	n	% in targets or background 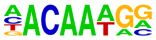		p-val
SOX2 unique	28,277	54.66%	31.71%	1e-1377
SOX2 / S-ctESRRB shared	56,293	47.32%	30.11%	1e-1591
SOX2 / S-ctESRRB unique	120,833	38.05%	25.92%	1e-1853

Fig. S10. Hybrid SOX2 factors bind new targets yet retain motif specificity. (A-B) HOMER motif scanning for SOX motifs in peak sets bound by **(A)** SOX2 and/or ntTCF1-SOX2 and **(B)** SOX2 and/or SOX2-ctESRRB.

Katznelson et al., Supplemental Figure 11

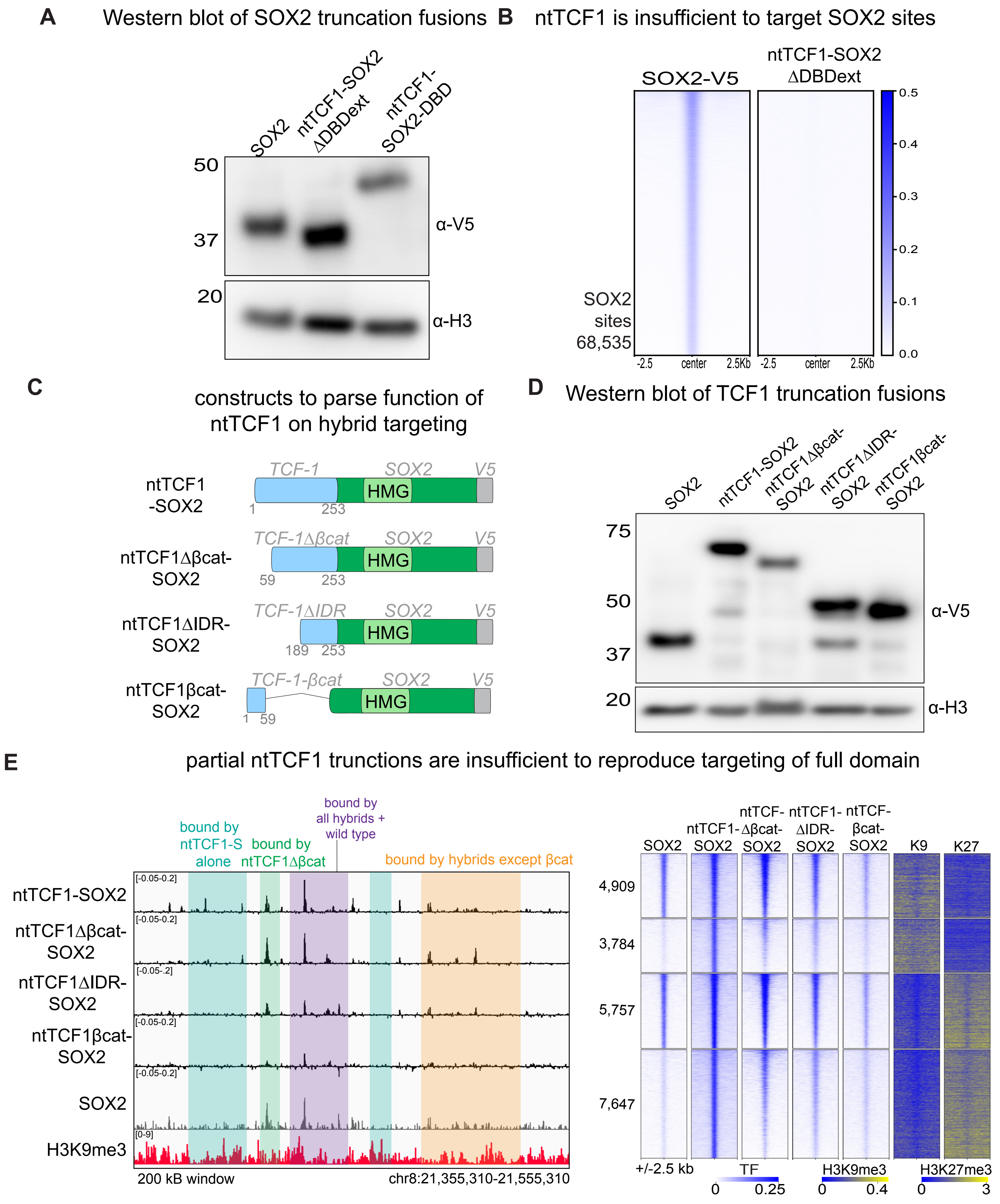

Fig. S11. Redundant non-DBD domains contribute to ntTCF1-SOX2 chromatin binding. **(A)** Western blot of V5-tagged SOX2, ntTCF1 fused to SOX2 with an extended DBD truncation, or the SOX2 DBD without non-DBDs. **(B)** Heatmap of ntTCF1-SOX2ΔDBDext over SOX2 binding sites. **(C)** Constructs to parse function of the ntTCF1 domain on hybrid targeting. **(D)** Western blot of ntTCF1-SOX2 truncation fusion proteins. **(E)** Example browser and heatmap views to demonstrate multiple non-redundant components of nTCF1 are required to fully enhance SOX2 targeting.

Katznelson et al., Supplemental Figure 12

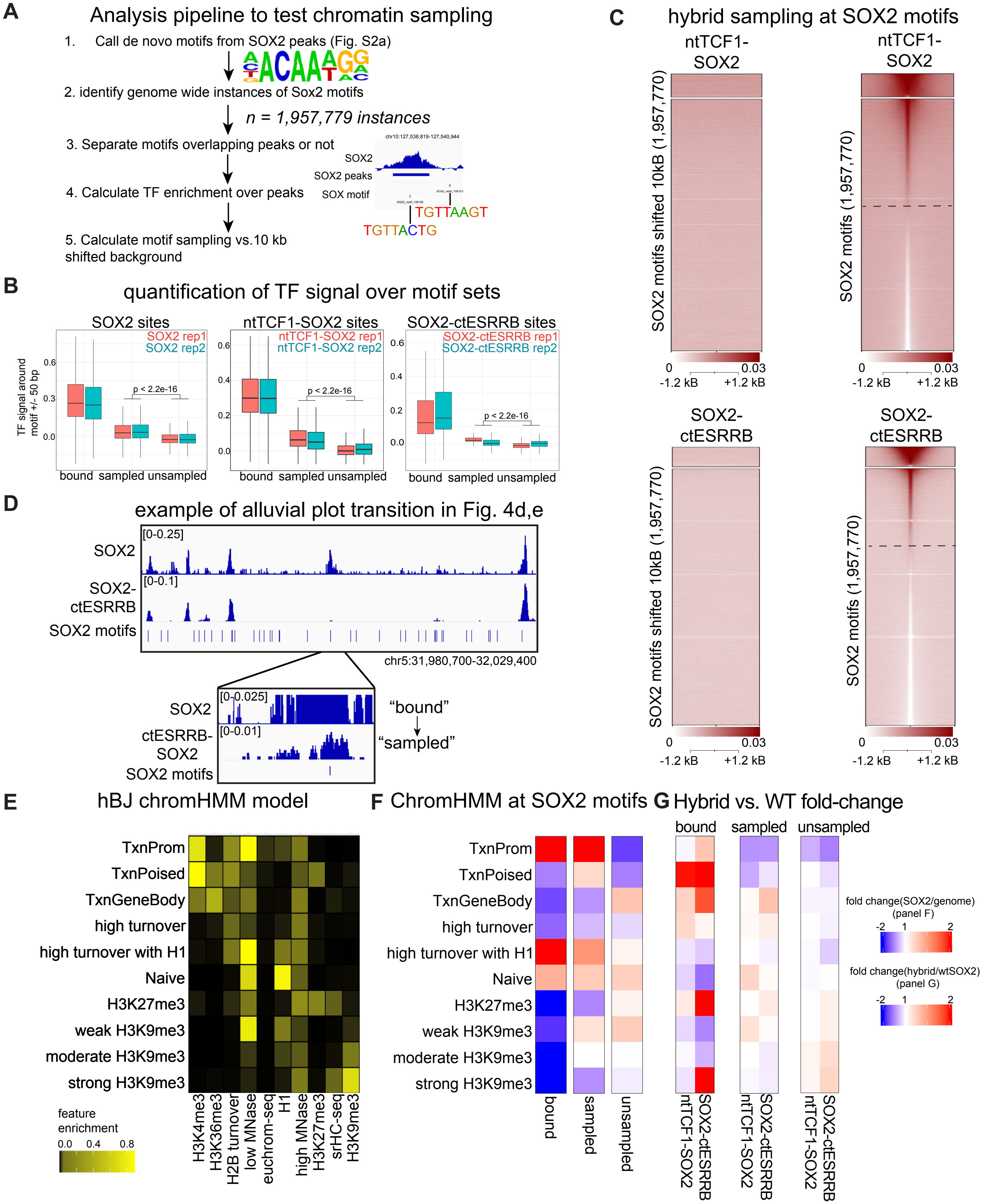

Fig. S12. Chromatin sampling by SOX2 hybrid proteins. (A) Analysis pipeline to assess chromatin sampling of SOX2 over its cognate motif. **(B)** Quantification of biological replicates of SOX2, ntTCF1-SOX2 and SOX2-ctESRRB over bound, sampled, or unsampled sites, $p < 2.2e-16$ as determined by Mann-Whitney U test. **(C)** Sampling of SOX2 motifs by ntTCF1-SOX2 (top) and SOX2-ctESRRB (bottom). Putative background sites, motif coordinates shifted 10 kB upstream, positioned to the left of each heatmap. **(D)** Genome browser region showing transition of a “bound” to “sampled” motif, as summarized in Fig. 4d and e. **(E)** 10-state chromHMM model from BJ fibroblasts. Feature annotations by manual assessment of chromatin feature enrichment and known combinatorial epigenetic modifications at different chromatin states. **(F)** chromHMM feature enrichment at SOX2 motifs either bound, sampled, or unsampled by wild type SOX2. **(G)** Enrichment fold change of SOX2 motifs bound sampled, or unsampled by SOX2 hybrids compared to wild type SOX2.

Katznelson et al., Supplemental Figure 13

A 48 hour ntTCF1-SOX2 (left) and SOX2-ESRRBct (right) over embryonic SOX2 targets

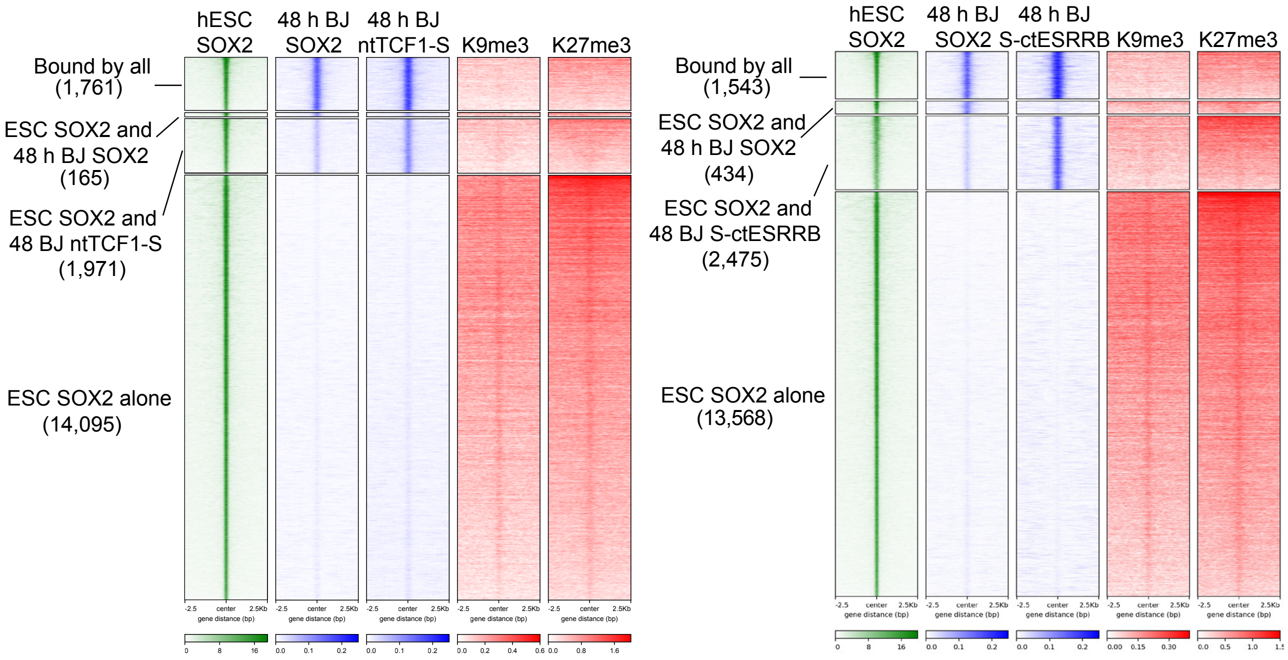

B GO analysis of genes targeted by ntTCF1-SOX2 peak subset

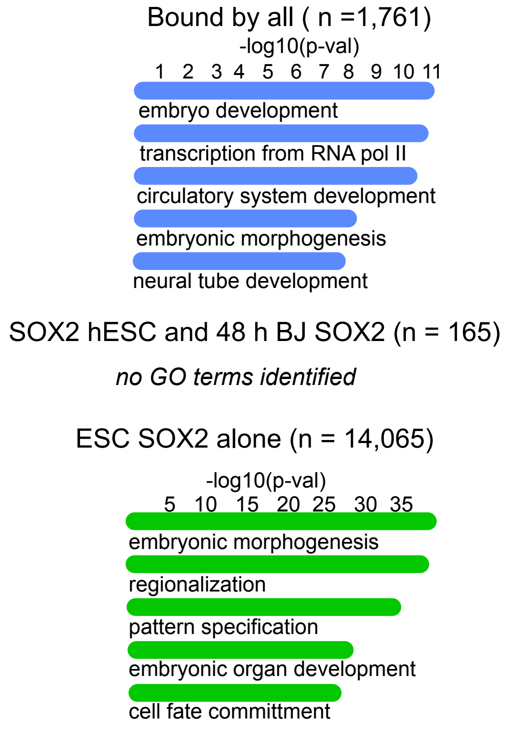

C GO analysis of genes targeted by SOX2-ctESRRB peaks

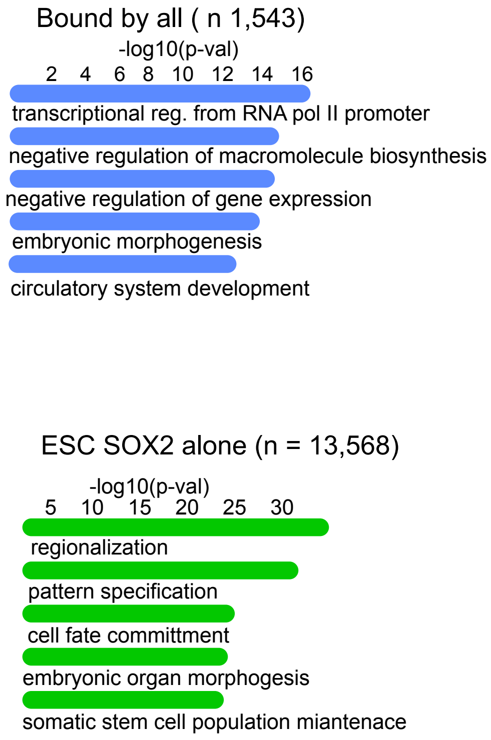

Fig. S13. Initial embryonic targets of SOX2 and ntTCF1-SOX2. (A) Heatmap of 48 h expressed SOX2, SOX2 hybrids over SOX2 binding sites in ESCs. **(B-C)** Gene ontology for genes within 50 kB of binding sites for **(B)** ntTCF1-SOX2 peak subsets and **(C)** SOX2-ctESRRB peak subsets.

Katznelson et al., Supplemental Figure 14

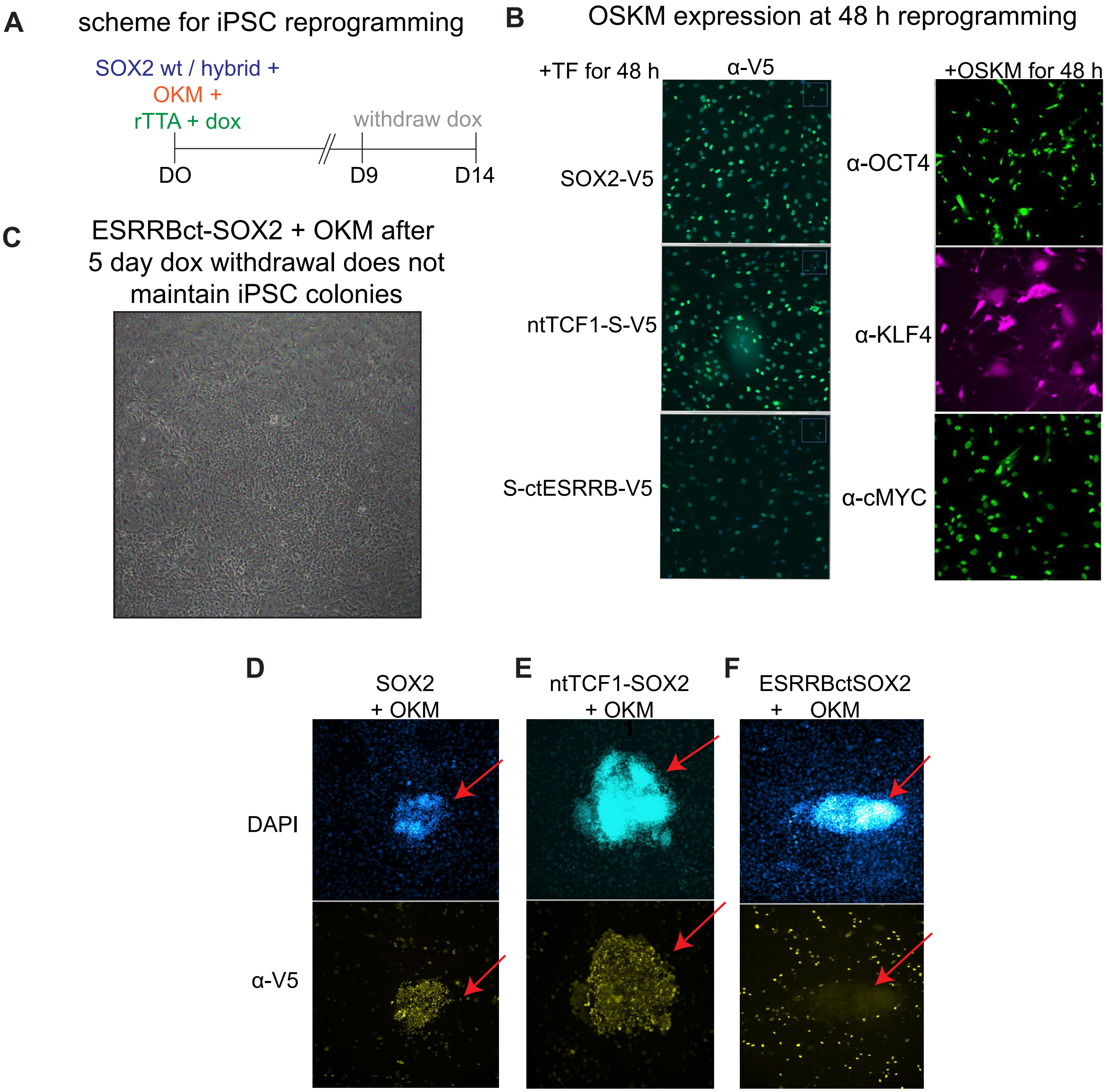

Fig. S14. iPSC reprogramming via lentiviral transduction. (A) Scheme for iPSC reprogramming via individual lentiviral transduction. **(B)** Immunofluorescence of anti-V5, anti-OCT4, anti-KLF4, anti-MYC in fibroblasts transduced for 48 hours with iPSC reprogramming lentiviral cocktail. **(C)** Brightfield image of MEFs transduced with SOX2-ctESRRB day 14 of iPSC reprogramming, 5 days post dox withdrawal. **(D-F)** Immunostaining of colonies at D9 of iPSC reprogramming finds expression of **(D)** wild type SOX2-V5, **(E)** ntTCF1-SOX2, and **(F)** silencing of SOX2-ctESRRB.

Table S1. Data origin for comparative transcription factor analysis

Transcription factor	ChIP antigen	expression	source
ASCL1	V5	48 h lentiviral in hBJ	This study
EBF1	V5	48 h lentiviral in hBJ	This study
EOMES	V5	48 h lentiviral in hBJ	This study
ESRRB	V5	48 h lentiviral in hBJ	This study
FOXA1	HALO	48 h lentiviral in hBJ	Lerner et al., 2023 (*4*)
GATA4	V5	48 h lentiviral in hBJ	Donaghey et al., 2019 (*23*)
HNF4A	HALO	48 h lentiviral in hBJ	Lerner et al., 2023 (*4*)
PDX1	V5	48 h lentiviral in hBJ	This study
PAX3	V5	48 h lentiviral in hBJ	This study
PAX7	V5	48 h lentiviral in hBJ	This study
RUNX1	V5	48 h lentiviral in hBJ	This study
SOX2	V5	48 h lentiviral in hBJ	This study
TCF-1	V5	48 h lentiviral in hBJ	This study

Table S2. Uniprot sequences used for gBlock design

Transcription factor	Accession number
ASCL1	P50553
EBF1	Q9UH73
EOMES	O95936
ESRRB	O95718
PDX1	P52945
PAX3	P23760
PAX7	P23759
RUNX1	Q2TAM6
SOX2	P48431
TCF-1	P36402

Table S3. All embryonic gene targets of SOX2 and SOX2 hybrids from Fig. S13
